# Antibacterial and Anti-Inflammatory Polysaccharide from *Fructus Ligustri Lucidi* Incorporated in PVA/Pectin Hydrogels Accelerate Wound Healing

**DOI:** 10.3390/molecules29071423

**Published:** 2024-03-22

**Authors:** Yanli Xi, Lianxin Hu, Xiang Chen, Lili Zuo, Xuesong Bai, Weijie Du, Na Xu

**Affiliations:** 1 Department of Toxicology, School of Public Health, Jilin Medical University, Jilin 132013, China; jilin2534126@163.com (Y.X.); 18755818472@163.com (X.C.); 17835600176@163.com (W.D.); 2 Department of Clinical Medicine, School of Clinical Medicine, Jilin Medical University, Jilin 132013, China; 13634445675@163.com; 3 Department of Food Quality and Safety, School of Public Health, Jilin Medical University, Jilin 132013, China; zuolili213@163.com; 4 Department of Nutrition, School of Public Health, Jilin Medical University, Jilin 132013, China; baixueso@163.com; 5 Office of Educational Administration, Jilin Medical University, Jilin 132013, China

**Keywords:** hydrogels, *Fructus Ligustri Lucidi* polysaccharides, polyvinyl alcohol, pectin, wound healing, antibacterial activity, anti-inflammatory

## Abstract

In cutaneous wound healing, an overproduction of inflammatory chemokines and bacterial infections impedes the process. Hydrogels can maintain a physiologically moist microenvironment, absorb chemokines, prevent bacterial infection, inhibit bacterial reproduction, and facilitate wound healing at a wound site. The development of hydrogels provides a novel treatment strategy for the entire wound repair process. Here, a series of *Fructus Ligustri Lucidi* polysaccharide extracts loaded with polyvinyl alcohol (PVA) and pectin hydrogels were successfully fabricated through the freeze–thaw method. A hydrogel containing a 1% mixing weight ratio of FLL-E (named PVA-P-FLL-E1) demonstrated excellent physicochemical properties such as swellability, water retention, degradability, porosity, 00drug release, transparency, and adhesive strength. Notably, this hydrogel exhibited minimal cytotoxicity. Moreover, the crosslinked hydrogel, PVA-P-FLL-E1, displayed multifunctional attributes, including significant antibacterial properties, earlier re-epithelialization, production of few inflammatory cells, the formation of collagen fibers, deposition of collagen I, and faster remodeling of the ECM. Consequently, the PVA-P-FLL-E1 hydrogel stands out as a promising wound dressing due to its superior formulation and enhanced healing effects in wound care.

## 1. Introduction

The postoperative use of wound dressings is crucial for restoring skin function. Currently, a wide array of dressings find application in managing chronic ulcers, burns, split graft donor sites, decubitus ulcers, and other related conditions [1]. It is imperative that wound dressings are used postoperatively in order for skin function to be restored [2]. In wound-dressing materials, hydrogels have garnered significant attention as targeted absorbents [3]. Pectin is widely used in wound dressings because it is hydrophilic, biocompatible, fairly inexpensive, relatively biodegradable, and has sound anti-inflammatory effects [4,5,6]. However, limitations exist regarding its resistance to moisture, mechanical strength, and barrier properties, necessitating the incorporation of reinforcing materials to enhance its physicochemical attributes [7]. Polyvinyl alcohol (PVA), known for its fiber/film-forming ability, chemical and mechanical resilience, non-carcinogenic nature, good barrier qualities, and high water-swelling capacity, is widely utilized [8,9,10]. However, for PVA to be used as a wound-dressing material, crosslinking is necessary, which makes it swell and retain water within its structure, but leaves it unable to dissolve in water [1,11]. Since PVA has a high absorption rate, it has been used to cure a variety of exudative lesions [12]. Pectin and PVA can also be used alone for hydrogel preparation, but their application in composite form eliminates the disadvantages of using them alone. PVA/pectin was designed as a biocompatible and non-toxic hydrogel and displayed its potential as a burn wound dressing or a bone regeneration scaffold [13,14]. Martinez et al. reported that the addition of pectin to PVA resulted in improved drug retention in the matrix [15]. Fishman and Coffin reported enhanced solubility of PVA/pectin blends as compared to PVA [16]. PVA/pectin-based blend nanofibers were reported as a novel material for wound healing applications [17]. Thus, PVA/pectin can be extensively used in drug delivery aids and wound-dressing materials [18]. Nevertheless, the direct use of PVA/pectin as a dressing matrix is infrequent due to its limited anti-inflammatory and antibacterial efficacy, which results in unsatisfactory wound healing [19]. It is reported that blending PVA/pectin with additional components may improve its mechanical performance and biodegradability [20,21]. 

*Fructus Ligustri Lucidi* (FLL), a traditional Chinese herb known as Nvzhenzi in Chinese, has been reported to exert antimicrobial action against *Staphylococcus aureus*, *Escherichia coli*, *Rhizopus*, *Aspergillus*, and *Bacillus subtilis* [22]. And the aqueous decoction and the ethanolic extract of FLL have a high inhibitory effect on bacteria [23]. The aqueous extract of FLL has anti-inflammatory and analgesic effects on mice [22]. In addition, treatment with an aqueous extract of FLL significantly reduced the torsional response to acetic acid, the pain from formalin, the swelling of feet, and the pain form lumbar disc herniation [23]. The significant amounts of arabinose, glucose, sucrose, rhamnose, and fucose present in FLL polysaccharides may be important contributors in supporting the immunological responses related to wounds [24,25]. It has also been proven that polysaccharide extract of FLL has antioxidative effects [26] and a low degree of side effects [22]. Antioxidation and low toxicity are related to the treatment of wound healing by dressing [27]. A natural FLL extract has antimicrobial, anti-inflammatory, analgesic, antioxidant, and low-toxicity properties, thus facilitating its clinical application in tissue engineering such as wound healing and visceral tissue repair. Therefore, incorporating FLL polysaccharide extracts (named FLL-E) into hydrogels to enhance their therapeutic effects without compromising their functionality seems promising for skin tissue engineering applications. However, to date, there have been no reported studies exploring the application of FLL-E in promoting the wound healing process. Thus, integrating FLL-E into PVA/pectin (named PVA-P) to create a wound-dressing product may hold promise as a valuable approach to investigate the impact of FLL-E on wound healing.

In this study, we fabricated PVA/pectin blend hydrogels containing FLL-E (named PVA-P-FLL-E) to promote wound healing. The overall objectives of the current study are to (i) determine the bioactive components present in the FLL seed extracts (FLL-E) prepared by a water extraction and alcohol precipitation method and diethylaminoethyl (DEAE) cellulose purification; (ii) evaluate the polysaccharide yield of the extracts using a phenol-sulfate method; (iii) prepare and characterize hydrogels containing FLL-E and PVA/pectin; (iv) assess the biological safety of PVA-P-FLL-E by an acute oral toxicity assay; (v) determine the wound healing properties of the dressings in a full-thickness excisional mouse model; and (vi) analyze the mechanism of PVA-P-FLL-E of wound healing promotion. 

## 2. Results 

### 2.1. Evaluation of FLL-E and PVA-P Hydrogel

High-performance liquid chromatography (HPLC) analysis demonstrated that FLL-E consisted of 11 monosaccharides and its molecular weight was 115156 Da (Figure S1a,b). The nuclear magnetic resonance (NMR) results indicated that FLL-E consisted acidic polysaccharides (Figure S1c). The characteristic analysis of PVA-P hydrogels showed that PVA: pectin (6:4) hydrogel has the best performance of all candidate hydrogels (Figures S2–S7). PVA: pectin (6:4) hydrogel was fixed for subsequent studies.

### 2.2. Evaluation of the PVA-P-FLL-E Hydrogels

**Scheme 1:**
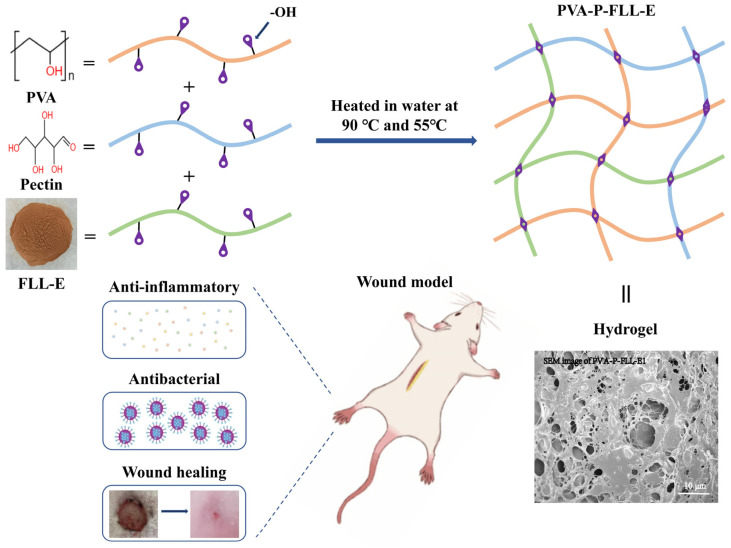
Schematic representation for the fabrication processes and application of PVA-P-FLL-E hydrogel.

**Figure 1:**
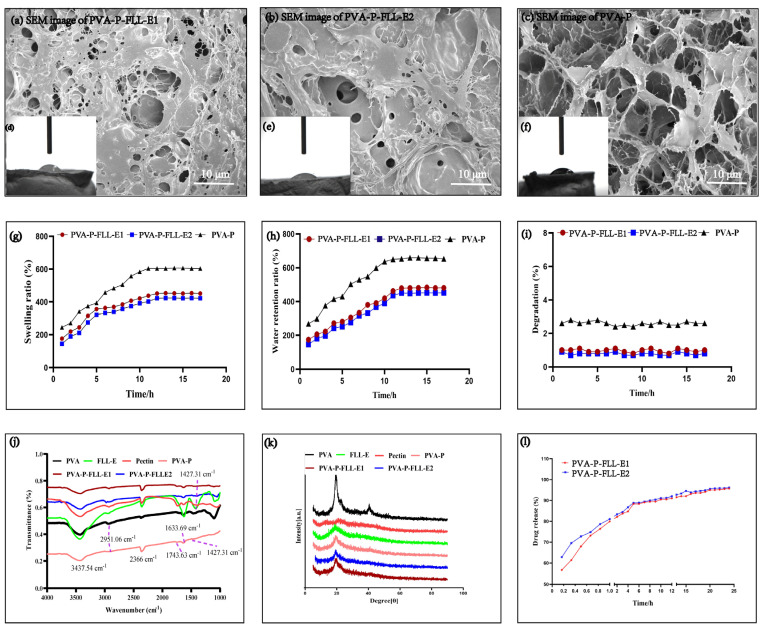
Morphological imaging (**a**–**c**) and contact angel (**d**–**f**) of the freeze-dried PVA-P-FLL-E1, PVA-P-FLL-E2, and PVA-P hydrogels. The swelling ratio (**g**), water retention ratio (**h**), and degradation ratio (**i**) as a function of time for PVA-P-FLL-E1, PVA-P-FLL-E2, and PVA-P hydrogels in PBS buffer from 1 to 24 h. Results represents the mean number of three samples tested to obtain a reliable value. Furthermore, the characterizations of all hydrogels were analyzed by FT-IR (**j**) and XRD (**k**). The percentage drug release as a function of time from the PVA-P-FLL-E1 and PVA-P-FLL-E2 hydrogels in PBS buffer at room temperature within 24 h (**l**). Results represent the mean number of three samples tested to obtain a reliable value.

Scheme 1 schematically illustrates the fabrication process of PVA-P-FLL-E hydrogels. SEM was employed to monitor the morphological properties of PVA-P-FLL-E1, PVA-P-FLL-E2, and PVA-P hydrogels. As shown in Figure 1a–c, the PVA-P hydrogels had irregular and interconnected porous microstructures. The numbers of micropores and porosity was slightly increased with the addition of FLL-E compared to PVA-P hydrogel (Figure S8a). As Figure 1d–f also show in the obtained values of contact angle, it was observed that the contact angle of the PVA-P-FLL-E hydrogels was slightly higher than that of PVA-P hydrogel, but there was no significant distinction between the PVA-P-FLL-E and PVA-P contact angle (*p* > 0.05, Supplementary Figure S8b). The concentration of FLL-E was positively correlated with the hydrophobicity of the hydrogel, indicating that the addition of FLL-E to the PVA-P led to an improvement in the hydrophobicity of the hydrogel.

The interaction between PVA-P and FLL-E contributed to the creation of a novel hydrophobic region, reinforcing the mechanical properties of the hydrogels. During the swelling test, all hydrogels exhibited swelling for up to 11 h, maintaining their swollen state thereafter (Figure 1g). The addition of FLL-E intensified hydrophobic interactions within the hydrogels, resulting in a lower SR and WR (Figure 1g,h). Specifically, the PVA-P hydrogel displayed a higher water uptake rate, swelling more than PVA-P-FLL-E1 and PVA-P-FLL-E2 hydrogels over time, ultimately reaching a higher final equilibrium. The decrease in equilibrium swelling observed for PVA-P-FLL-E1 and PVA-P-FLL-E2 hydrogels is attributed to the lower density of PVA chains resulting from the inclusion of pectin and FLL-E. This trend is also evident in the WR curves, displaying lower WR for PVA-P-FLL-E1 and PVA-P-FLL-E2 hydrogels compared to the PVA-P hydrogel. Notably, the SR and WR of the PVA-P-FLL2 hydrogel were lower than those of PVA-P-FLL-E1 due to its higher hydroxyl group content. Consequently, the PVA-P-FLL-E2 hydrogel reached equilibrium swelling more rapidly than PVA-P-FLL-E1. Additionally, results indicated that PVA-P-FLL-E1, PVA-P-FLL-E2, and PVA-P hydrogels exhibited comparable DR when stored at room temperature (Figure 1i).

As illustrated in Figure S8c, UV spectra analysis exhibited no detectable absorption peaks at 260 or 280 nm, confirming the absence of nucleic acids and proteins in FLL-E. Notably, UV spectra comparison revealed no significant disparities between PVA-P-FLL-E1 and PVA-P-FLL-E2 hydrogels. FT-IR spectra, depicted in Figure 1j, showcased predominant peaks related to PVA, pectin, and FLL-E. The peak at 3437.54 cm^−1^ corresponded to OH bond stretch vibrations, while 2951.06 cm^−1^ was associated with CH/CH_2_ bond stretches. A consistent oscillation band at 2366 cm^−1^ was evident across all samples. Notably, peaks around 1743.63, 1633.69, and 1427.31 cm^−1^ indicated the presence of -COOH vibrations, characteristic of acidic polysaccharides of FLL-E. This observation aligned with monosaccharide composition and NMR analysis, confirming the composition of FLL-E primarily consisting of acidic polysaccharides. Pectin is a linear polygalacturonic acid and polyL-rhamnose-galacturonic acid with hydroxyl group methylated to varying degrees. Indeed, the primary chemical groups present in the mentioned saccharides, the -OH, CH_2_, and C-O groups, are analogous to those found in the structure of PVA. Consequently, during FT-IR analysis, the vibration bands associated with these chemical groups in PVA-P-FLL-E1 and PVA-P-FLL-E2 demonstrated overlapping patterns with PVA. This absence of additional bands was specific to PVA-P-FLL-E1 and PVA-P-FLL-E2 hydrogels. The findings highlight the favorable compatibility achieved among FLL-E, pectin, and PVA, primarily facilitated by the formation of hydrogen bonds between their respective hydroxyl groups.

The XRD diffraction patterns are shown in Figure 1k. The XRD pattern for PVA presented typical semi-crystalline peaks, and PVA-P-FLL-E1, PVA-P-FLL-E2, and PVA-P hydrogels presented crystalline peaks at around 20°. In the PVA-P and PVA-P-FLL-E hydrogels, there were no significant differences between peaks, but in the PVA-P-FLL-E2 hydrogel, the percentage reduction in the peak intensities was highlighted. 

The ability to gradually and continuously deliver drugs can also accelerate the wound healing process [28]. Hence, a preliminary study of the in vitro release profiles of polysaccharide from the PVA-P-FLL-E hydrogels was evaluated in PBS pH7.4. PVA-P-FLL-E1 showed 67.96% of the accumulative release within only 30 mins (Figure 1l), while 95.70% of the release was measured over a duration of 24 h. Meanwhile, PVA-P-FLL-E2 showed 72.72% of the released mass of drug within 30 mins, and it released more than 96% of its payload in 24 h (Figure 1l).

The transparency of the hydrogel allows for direct observation of the healing process without the need for removing the dressing. The color of FLL-E powder is brown, and the color of the hydrogel is proportional to the FLL-E content. The addition of FLL-E enhanced the ΔE value of the hydrogels (Figure S9a). High concentration of FLL-E will affect the transparency of hydrogel.

Regarding adhesive strength (Figure S9b), the addition of FLL-E significantly enhanced the adhesive properties of PVA-P-FLL-E hydrogels. Particularly, the adhesive strength of PVA-P-FLL-E2 hydrogel surpassed that of PVA-P-FLL-E1. Previous studies proved that low-adhesive-strength hydrogels were easily detached from the skin surface [5]. This facilitates the replacement of wound dressings without causing secondary damage.

### 2.3. Antimicrobial Analysis

**Figure 2:**
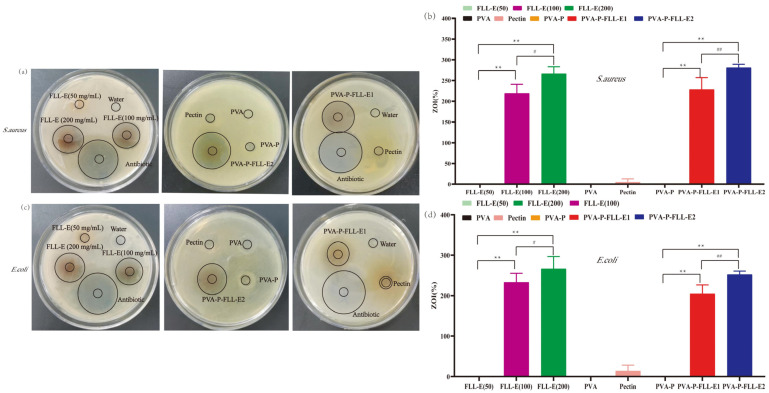
Antibacterial result of PVA-P-FLL-E hydrogels evaluated by Oxford cup test. Representative images of the inhibitory effect of *S. aureus* (**a**) and *E. coli* (**c**) on agar plate. The average ZOI graphs of *S. aureus* (**b**) and *E. coli* (**d**) after co-culture with hydrogels. FLL-E (50), FLL-E (100), and FLL-E (200) indicate 50 mg/mL, 100mg/mL, and 200 mg/mL of FLL-E were used to co-culture with *S. aureus* and *E. coli*. Data are presented as means ± SD. For FLL-E (50), FLL-E (100) and FLL-E (200) data, FLL-E (50) was set as control. For PVA, PVA-P-FLL-E1 and PVA-P-FLL-E2 data, PVA was set as control. Statistical significance: ** *p* < 0.01; ^#^ *p* < 0.05, ^##^ *p* < 0.01.

Figure 2 shows the antibacterial activity of hydrogels against *Staphylococcus aureus* (*S. aureus*, Gram-positive bacteria) and *Escherichia coli* (*E. coli*, Gram-negative bacteria). As shown in Figure 2a,b, the diameter of the inhibition zone and the average ZOI of *S. aureus* were increased with the increase in FLL-E concentrations. Notably, the sensitivity of *S. aureus* to FLL-E was slightly higher compared to *E. coli*, but FLL-E still had good antibacterial activity against *E. coli* (Figure 2c,d). Further exploration into the impact of PVA and pectin on the ZOI of *S. aureus* and *E. coli* revealed insightful findings. Pectin demonstrated a ZOI of 4.76 ± 2.25% in *S. aureus* plates and 14.29 ± 2.25% in *E. coli* agar plates, indicating limited antibacterial activity. Conversely, PVA and blank controls showed no observable ZOI. The ZOI of PVA-P hydrogel was smaller than that of the pectin. However, compared with PVA-P hydrogel, PVA-P-FLL-E hydrogels displayed notable antibacterial activity against both *S. aureus* and *E. coli* (*p* < 0.01). Additionally, similar to FLL-E, the bactericidal ability of PVA-P-FLL-E hydrogels on *S. aureus* and *E. coli* was significantly improved with the addition of FLL-E. These findings suggest that the antibacterial properties of PVA-P-FLL-E hydrogels primarily stem from the controlled release of FLL-E. This controlled release mechanism holds promise for sustained antibacterial activity. Importantly, a substantial difference in ZOI was observed between PVA-P-FLL-E2 and PVA-P-FLL-E1 hydrogels (*p* < 0.01), with PVA-P-FLL-E2 demonstrating significantly higher ZOI, indicating the dosage-dependent effect of FLL-E on antibacterial efficacy.

Although the performance of PVA-P-FLL-E2 is better than that of PVA-P-FLL-E1, the antibacterial concentration of PVA-P-FLL-E1 is smaller than that of PVA-P-FLL-E2. Moreover, our results clearly indicated that the PVA-P-FLL-E1 had excellent antibacterial activity against *S. aureus* and *E. coli.* In order to avoid overmedication, PVA-P-FLL-E1 is selected for subsequent experiments.

### 2.4. PVA-P-FLL-E1 Hydrogels Promoted Skin Wound Healing 

**Figure 3:**
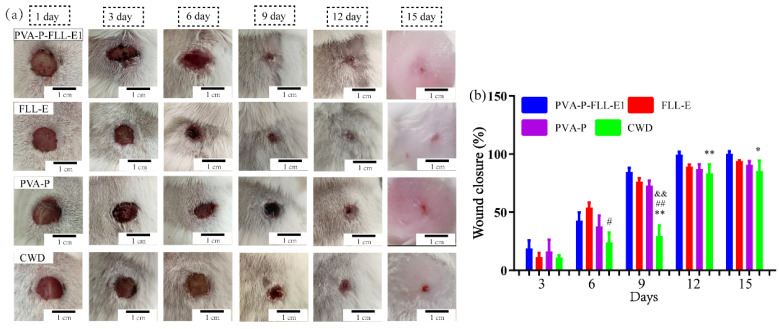
Effect of PVA-P-FLL-E1 hydrogel on full-thickness cutaneous wound model of murine dorsum. Representative images of the wounds pretreated with PVA-P-FLL-E1, PVA-P, FLL-E, and CWD (**a**). The wound healing rate versus healing time (on the 1st, 3rd, 6th, 9th, 12th, and 15th day after surgery) in each group (**b**). Scale bars: 100 mm. Error bars indicate SD. Significant differences between sample means are indicated; * *p* < 0.05, ** *p* < 0.01 versus PVA-P-FLL-E1 group; ^#^ *p* < 0.05, ^##^ *p* < 0.01 versus FLL-E group; ^&&^ *p* < 0.01 versus PVA-P group.

The prepared samples were applied at the wound sites, and then wound images for each time point (1, 3, 6, 9, 12, and 15 days) were captured, as shown in Figure 3a. On the first day post-treatment, slight inflammation and swelling were visible around the wound areas across all groups. On the third day post-operation, there was no evidence of necrosis in any wound surfaces of the mice. Due to the anti-inflammatory properties of FLL-E, animals treated with PVA-P-FLL-E1 displayed dried wound surfaces and the formation of scabs, while those treated with FLL-E and CWD (negative control) still exhibited inflammation and exudates. On the sixth day after surgery, all groups showed reduced wound areas on average, with the PVA-P-FLL-E1 hydrogel showing the most significant reduction in wound areas. Moreover, there was a noticeable decrease in swelling around the wound area in all animals compared to the first and third days. At the end of the nineth day post treatment, scabs were clearly evident in mice in the CWD and FLL-E groups. The PVA-P-FLL-E1 and PVA-P hydrogels, however, barely exhibited scabs. On the 12th day post-treatment, the PVA-P-FLL-E1 group exhibited substantial healing progress, displaying thickening of the epidermal layer. From the 15th day post-operation, the majority of wounds in the PVA-P-FLL-E1 group appeared almost completely healed, with the epidermis sealing and showing signs of complete closure.

The measurement of wound areas in mice treated with various materials allowed for the calculation of wound healing rates during the observation period. As shown in Figure 3b, on day 9, the wound healing rate in the CWD group was (29.44 ± 9.23)%, which was significantly increased to (84.66 ± 3.43)% in the PVA-P-FLL-E1 group, (76.36 ± 2.80)% in the FLL-E group, and (72.92 ± 4.32)% in the PVA-P group (*p* < 0.01). On the 15th day, the wound healing rate in the PVA-P-FLL-E1 group neared 100%, but that of CWD group was only (85.43 ± 8.93)%. As expected, the best material for wound healing was PVA-P-FLL-E1. 

### 2.5. PVA-P-FLL-E1 Hydrogels Promoted Wound Construction

**Figure 4:**
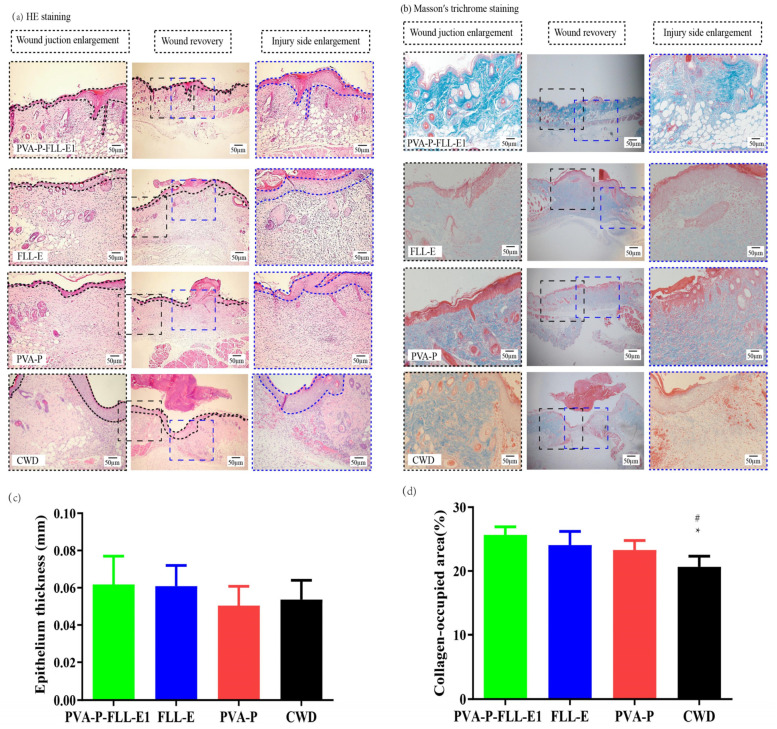
Images of the skin morphology obtained with H&E staining (**a**) and Masson′s trichrome (MTC) staining in (**b**) sections of the tissues on the injured side (blue dotted frame) and wound junction (black dotted frame) after 15 days of wound healing and their corresponding enlarged images. The average epithelium thickness of the wounds (**c**) and density of collagen fibers (**d**) were calculated on the basis of H&E- or MTC-stained images. Error bars indicate SD. The magnifications are ×40 and ×200, respectively. Significant differences between sample means are indicated; * *p* < 0.05 versus PVA-P-FLL-E1 group; ^#^ *p* < 0.05 versus FLL-E group.

The comparison between PVA-P-FLL-E1 hydrogel and CWD revealed notable differences in wound healing and tissue regeneration. As shown in Figure 4a, re-epithelialization of desquamated epithelial regions was notably more extensive in the PVA-P-FLL-E1 hydrogel-treated group than in the CWD-treated group. The PVA-P-FLL-E1 hydrogel demonstrated reduced inflammatory cell infiltration, increased neovascularization in granulation tissues, and a higher presence of collagen proliferation compared to CWD-treated wounds. The epidermis thickness of regenerated skin was slightly greater after PVA-P-FLL-E1 treatment when compared to the other treatments, but they did not show significance differences (Figure 4c, *p* > 0.05). Moreover, the PVA-P-FLL-E1 hydrogel notably facilitated re-epithelialization and skin tissue reconstruction on full-thickness wounds compared to CWD, as evident from MTC staining (Figure 4b). The wound treated with PVA-P-FLL-E1 showed dense and regular collagenous fibers, while the fibroblast arrangement of the wound area in the CWD group was disordered. None of the wounds in the PVA-P-FLL-E1 group exhibited any evidence of necrosis, edema, or abscess. The PVA-P-FLL-E1 group showed higher collagen-occupied area as compared to skin tissue of the CWD group (*p* < 0.05, Figure 4d). The maximum collagen-occupied area was observed in mice protected by PVA-P-FLL-E1 hydrogel. This could be attributed to the characteristics of the obtained hydrogel.

### 2.6. PVA-P-FLL-E1 Hydrogels Enhanced the Collagen Deposition

**Figure 5:**
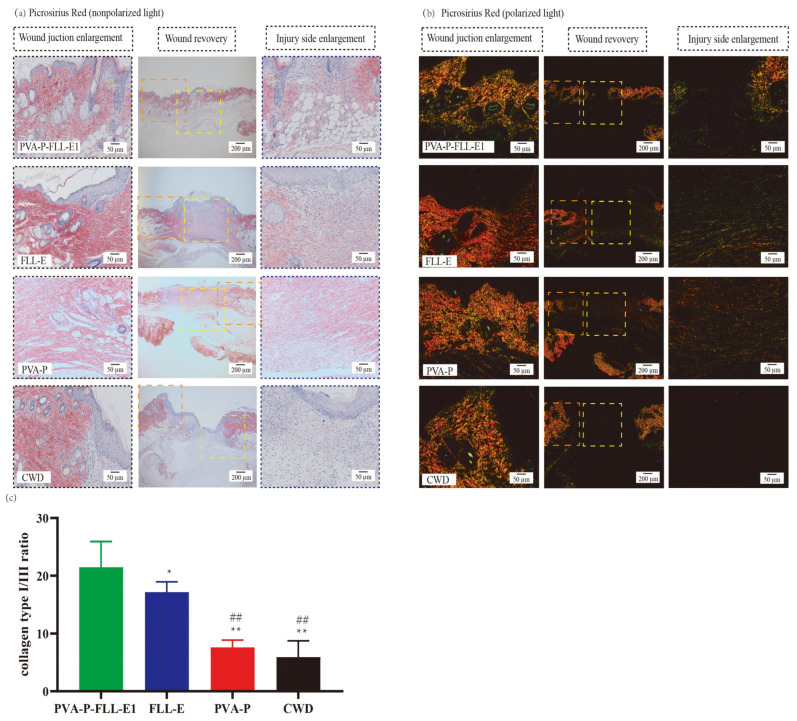
Representative nonpolarized (**a**) and polarized (**b**) photomicrographs (PSR staining) of the PVA-P-FLL-E1, FLL-E, PVA-P, and CWD groups, at the 15th day after wound induction; rectangular box represents the sections of tissues on the injured side (yellow dotted frame) and wound junction (orange dotted frame) and their corresponding enlarged images, (**c**) showing the percentage of increase in the number of collagen fibers after PVA-P-FLL-E1 treatments. Error bars indicate SD. The magnifications are ×40 and ×200, respectively. Significant differences between sample means are indicated; ** *p* < 0.01, * *p* < 0.05 versus PVA-P-FLL-E1 group; ^##^ *p* < 0.01 versus FLL-E group.

The analysis of collagen type I–collagen type III ratio, conducted via PSR staining (Figure 5a,b), revealed significant findings. The ratio between collagen type I and collagen type III within the wound area was significantly higher in the PVA-P-FLL-E1 hydrogel-treated group compared to the other three groups on the 15th day (*p* < 0.05, *p* < 0.01, Figure 5c). Additionally, an increase in the ratio of collagen type I to collagen type III was observed in the FLL-E group in comparison to the CWD group (*p* < 0.01, Figure 5c). The PVA-P-FLL-E1 hydrogel greatly enhanced the collagen deposition.

### 2.7. PVA-P-FLL-E1 Improved the Immune Microenvironment 

**Figure 6:**
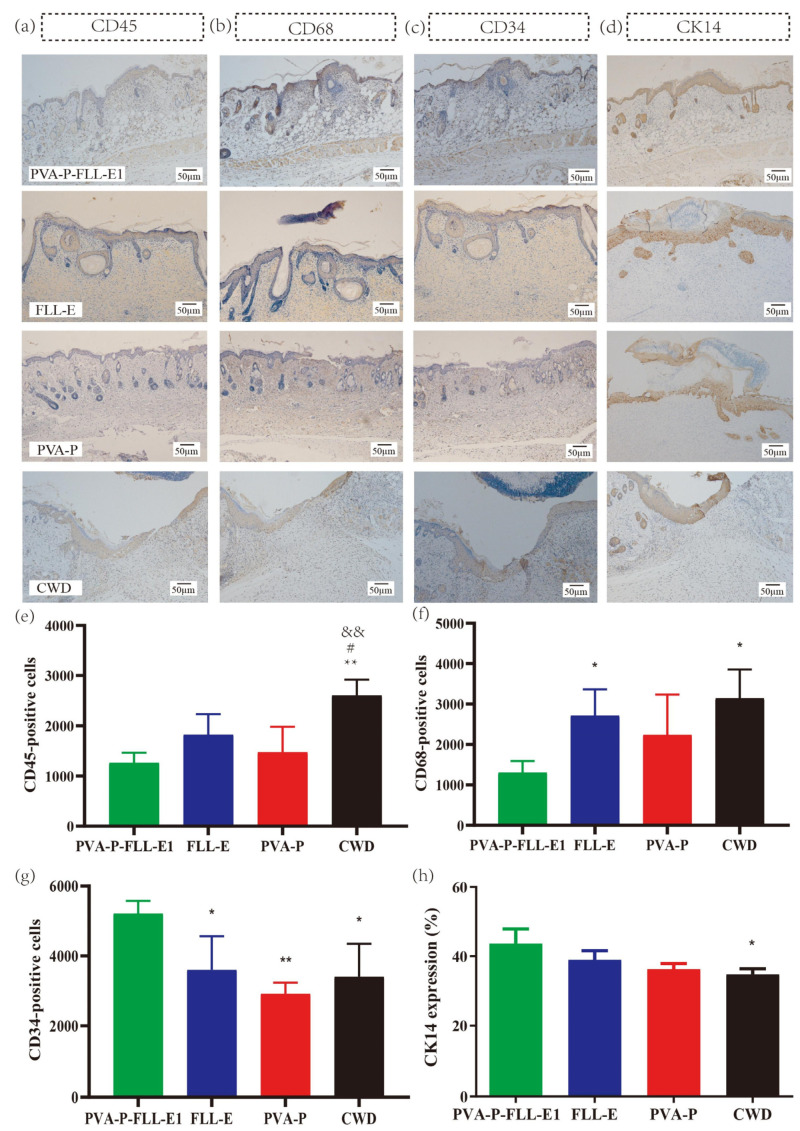
Immunohistochemical evaluation and quantitative analysis of the density of positive staining at murine wound sites. On the 15th day after full-thickness skin incision creation, different markers were tested for the presence of CD45 (**a**), CD68 (**b**), CD34 (**c**), and CK14 (**d**). The density of positive staining of CD45 (**e**), CD68 (**f**), CD34 (**g**), and CK14 (**h**) was analyzed by Image J 1.53e software. The study utilized five tissue sections from each experimental group, with five distinct fields analyzed within each section through specialized software. Error bars indicate SD. Statistical significance indicating differences between sample means has been indicated; ** *p* < 0.01, * *p* < 0.05 versus PVA-P-FLL-E1 group; ^#^ *p* < 0.05 versus FLL-E group; ^&&^ *p* < 0.01 versus PVA-P group. Scale bars 50 μm. The magnification is ×100.

From our findings, robust CD45 staining was detected in the wound tissues of all groups, which is an identification marker of leukocytes in the inflammatory process (Figure 6a). Leukocyte increase is related to inflammation response. Remarkably, the PVA-P-FLL-E1 hydrogel exhibited a significant reduction in CD45 expression compared to the CWD group (*p* < 0.01, Figure 6e). This suggests that one of the crucial mechanisms by which the PVA-P-FLL-E1 hydrogel promotes wound healing is by inhibiting the inflammatory response. The macrophage lineage-specific protein CD68 can be used for assessing the number of macrophages infiltrating a wound healing area. Macrophages can respond to infection by enhancing the immune response, and the initial phenotype of macrophages in wound healing is inflammatory. The evaluation of CD68 revealed positive reactions in all four groups, signifying inflammatory responses during wound healing (Figure 6b). There is a dramatic decrease in CD 68 expression at the wound sites treated by PVA-P-FLL-E1 hydrogel compared with the FLL-E-treated wound sites (*p* < 0.05). Uniformly, the PVA-P-FLL-E1 group showed less CD68 immune reaction than the CWD group during wound healing process (*p* < 0.05, Figure 6f). 

### 2.8. PVA-P-FLL-E1 Hydrogels Regulated the Re-Epithelialization Process 

CD34 intense staining indicated a process of angiogenesis during the wound closure process. Further staining indicated that wound sites treated by PVA-P-FLL-E1 hydrogel were strongly expressing CD34 markers (Figure 6c). The CD34-positive expression level in the PVA-P-FLL-E1 group was notably the highest, displaying statistical differences compared to the other three groups (*p* < 0.05, *p* < 0.01, Figure 6g). These findings strongly suggest that PVA-P-FLL-E1 effectively promotes angiogenesis, which plays a pivotal role in facilitating tissue remodeling within the wound area. Furthermore, the wound tissue sections treated with the PVA-P-FLL-E1 hydrogel were analyzed for CK14 staining, a characteristic marker of basal keratinocytes responsible for constructing a protective barrier against the external environment. Brown staining is considered as CK14-positive, and it was observed in all groups (Figure 6d). The PVA-P-FLL-E1 hydrogel significantly improved CK14 expression in epithelial cells compared with CWD, indicating that it can promote keratinocyte differentiation and migration (*p* < 0.05, Figure 6h).

### 2.9. PVA-P-FLL-E1 Hydrogels Were Involved in Decreasing the Wound Inflammatory Response

**Figure 7:**
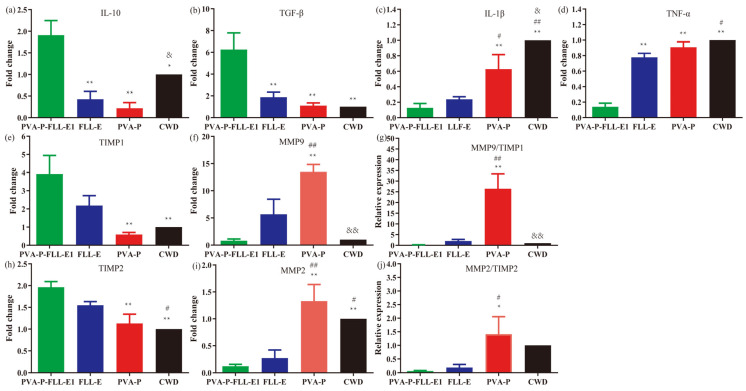
PVA-P-FLL-E1 upregulates IL-10, TGF-β, TIMP1, TIMP2, and downregulates IL-1β, TNF-α, MMP9, MMP2, MMP9/TIMP1, MMP2/TIMP2 in response to the degree of inflammatory reaction on day 15 after full-thickness skin incision creation. Changes in IL-10 (**a**), TGF-β (**b**), IL-1β (**c**), TNF-α (**d**), TIMP1 (**e**), MMP9 (**f**), MMP9/TIMP1 (**g**), TIMP2 (**h**), MMP2 (**i**), MMP2/TIMP2 (**j**) in all groups analyzed by qRT-PCR method. Error bars indicate SD. Significant differences between sample means are indicated; * *p* < 0.05, ** *p* < 0.01 versus PVA-P-FLL-E1 group; ^#^ *p* < 0.05, ^##^ *p* < 0.01 versus FLL-E group; ^&^ *p* < 0.05, ^&&^ *p* < 0.01 versus PVA-P group.

qRT-PCR analysis was employed to assess mRNA expression levels of anti-inflammatory cytokines (IL-10 and TGF-β) and pro-inflammatory cytokines (TNF-α and IL-1β) within wound tissues from each group on the 15th day post-surgery. The mRNA levels of anti-inflammatory cytokines were notably lower in the FLL-E, PVA-P, and CWD groups compared to the PVA-P-FLL-E1 group (*p* < 0.05, *p* < 0.01, Figure 7a,b). Specifically, the relative IL-10 mRNA level in the CWD group exhibited a significant increase compared to the PVA-P group (*p* < 0.05, Figure 7a). Additionally, the mRNA levels of pro-inflammatory cytokines were significantly elevated in the PVA-P group compared to the PVA-P-FLL-E1 group (*p* < 0.01). Furthermore, the relative levels in the CWD group were increased compared to both the PVA-P-FLL-E1 and PVA-P groups (*p* < 0.05, *p* < 0.01, Figure 7c,d).

### 2.10. PVA-P-FLL-E1 Hydrogels Promoted ECM Remodeling

We next measured mRNA levels of MMP2, MMP9, TIMP1, and TIMP2 in PVA-P-FLL-E1-treated wound tissues and compared them with CWD. The expression of MMP2 and MMP9 was notably downregulated in the PVA-P-FLL-E1- or FLL-E-treated animals in comparison to the PVA-P group (*p* < 0.01). Conversely, the expression of TIMP1 and TIMP2, known inhibitors of MMP2 and MMP9, respectively, showed significant upregulation in the PVA-P-FLL-E1-treated tissue sections compared to the normal wounds in the CWD group (*p* < 0.01, Figure 7e,f,h,i). Moreover, the MMP9/TIMP1 and MMP2/TIMP2 ratios were significantly lower in the PVA-P-FLL-E1 group compared with the PVA-P group (*p* < 0.05, *p* < 0.01, Figure 7g,j). 

## 3. Materials and Methods

### 3.1. Chemicals 

The chemicals and reagents utilized throughout this study were of analytical grade. The solvent, polyvinyl alcohol (PVA, Mw: 440,000, 98% hydrolyzed), was purchased from Sigma, Ronkonkoma, NY, USA. Pectin (Mw: 50,000~300,000, degree of esterification of 65–70%, from citrus peel) was purchased from Sangon Biotech (Shanghai) Co., Ltd., Shanghai, China. All other chemicals were obtained from Beijing Sinopharm Chemical (Beijing, China). *Escherichia coli* ATCC 25922 and *Staphylococcus aureus* ATCC 25923 (from the China General Microbiological Culture Collection Center (CGMCC), Beijing, China) were cultured according to manufacturer’s requirements [29]. 

### 3.2. Collection and Preparation of Active Ingredients

*Fructus Ligustri Lucidi* (FLL), sourced as the dried mature fruit of *Ligustrum lucidum* Ait., was purchased from Tongrentang Pharmaceutical Co., Ltd., Beijing, China. The extraction of active ingredients from FLL was conducted via hot water extraction, following a previously outlined method with slight modifications [30]. Detailed extraction steps are provided in the Supplementary data.

### 3.3. Preparation of PVA-P Hydrogels

PVA-P hydrogels with different mass ratios of PVA and pectin (2:8, 4:6, 5:5, 6:4, 8:2, *w*/*w*) were prepared by the freezing–thawing cycle. A total of 10 g of the above powder was dissolved in distilled water to form a 10% concentration solution. Then the solution was stirred at 90 °C at 1400 rpm for 2 h before being transferred to a −80 °C refrigerator. To achieve physical cross-linking, the samples were frozen at −80 °C for 18 h, and thawed at room temperature for 2 h to 3 h. These procedures were repeated three times. Finally, the hydrogel was freeze-dried to form air-dried films. The characterizations of hydrogel were analyzed to select the most suitable dressing for wounds.

### 3.4. Preparation of PVA-P-FLL-E Hydrogels 

The process began by preparing an aqueous solution of PVA/pectin (PVA-P) at a concentration of 10% (*w*/*v*). This solution was created through continuous stirring at 90 °C and 1400 rpm for 2 h. Following this, solutions containing either 1% or 2% (*w*/*v*) of FLL-E were added to the PVA-P solution. The stirring continued at 1000 rpm for 1 h at 55 °C to ensure a homogeneous mixture, resulting in the formation of hydrogels named PVA-P-FLL-E1 and PVA-PFLL-E2. Equal weights of PVA-P, PVA-P-FLL-E1, and PVA-P-FLL-E2 hydrogels were cast into Petri dishes. These hydrogels were freeze-dried to form air-dried films. Before surgery, the dried hydrogel was submerged in distilled water for 30 min. Thickness was evaluated on 5 random spots per sample using a spiral micrometer (DL321025B, Deli Co., Ltd., Ningbo, China). 

### 3.5. Characterization of the Hydrogels

The surface morphology of the PVA-P-FLL-E1, PVA-P-FLL-E2 and PVA-P hydrogels was examined by scanning electron microscope (SEM) in a JSM6010 LA (JEOL, Akishima-shi, Japan) after gold deposition at a magnification of 2000 in magnitude using secondary electrons, under low-vacuum conditions between 3 kV and 15 kV. Porosity was calculated based on Supplementary Equation S(1). Three dried samples of the PVA-P-FLL-E1, PVA-P-FLL-E2, and PVA-P hydrogels were measured using a contact angle goniometer (Kyowa Interface Science, Niiza, Japan) at room temperature. The swelling ratio (SR), water retention ratio (WR), and degradation ratio (DR) of the PVA-P-FLL-E1, PVA-P-FLL-E2, and PVA-P hydrogels were determined by weighing the wet and dry samples, which were calculated based on Supplementary Equation S(2). Then, Fourier transform infrared spectroscopy (FT-IR) was performed using a Nicolet iS5 (Thermo Fisher Scientific, Waltham, MA, USA) spectrometer to analyze the structures of the hydrogels at a wave number between 4000 and 400 cm^−1^. X-Ray diffractometry (XRD) using X’Pert PR (PANalytical B.V., Almelo, The Netherlands) equipment and UV spectra analysis via an Evolution 201 spectrometer (Thermo Fisher, Waltham, MA, USA) were also conducted. Color determination was carried out on five random zones per sample using YS3060 (Shanenshi Co., Ltd., Shenzhen, China). Finally, adhesive strength was determined using a texture analyzer (TA.XTC-18, Shanghai baosheng Co., Ltd., Shanghai, China).

### 3.6. Drug Release Measurement

In brief, 10 mL of PBS (pH 7.4, 37 °C) was mixed with 0.1 g PVA-P-FLL-E in freeze-dried condition, and release of polysaccharide from PVA-P-FLL-E hydrogels was determined by the phenol-sulfate method, as previously reported [31]. A total of 1 mL solution of samples in PBS was withdrawn at recorded time points and 1 mL PBS was added to keep the total amount of solution at 10 mL. Tests were carried out in triplicate.

### 3.7. Antimicrobial Analysis

The Oxford cup method was used to measure the antibacterial ability [29]. *S. aureus* and *E. coli* were grown in LB solid medium at 37 °C for 24 h and a single colony was transferred into 5 mL of fresh LB liquid medium at 37 °C for 24 h with shaking. In all, 100 μL bacterial suspension was evenly spread on the pre-prepared solid LB agar culture using an applicator. Each Oxford cup, with an inner diameter of 7 mm, was filled with 100 μL of the sample, antibiotic solution (positive control), or sterile water (negative control) and placed at the center of the agar plate. After incubation at 37 °C for 24 h, the plates were observed for inhibition zones. This process was replicated three times for each concentration, and the zone of inhibition (ZOI) was measured using Supplementary Equation S(3).

### 3.8. In Vivo Assay 

#### 3.8.1. Animals

The in vivo experiment adhered to the Animal Management Rule of the Ministry of Public Health, China, and all the procedures were previously approved by the Ethics Committee of Jilin Medical University, Jilin, China (2023-LW001). Male ICR mice were used with a body weight around 18–22 g, and they were purchased from Changsheng Biotechnology Co., Ltd. (License number: SCXK(Liao)2020-0001, Shenyang, China. No.210726210101302823). All mice were individually housed in polycarbonate cages under controlled conditions: 23 ± 2 °C temperature, 50 ± 10% humidity, and a 12 h light–dark cycle, with ad libitum access to standard laboratory food (pellet diet) and tap water.

#### 3.8.2. Experimental Protocol

The animals underwent a week-long acclimatization period in the laboratory environment prior to the commencement of the experiment. A full-thickness excisional model was employed to generate the wounds. Anesthetization was achieved via an intraperitoneal injection of 2% pentobarbital (45 mg/kg body weight). The dorsal hair was shaved using an electric razor, followed by the application of a depilatory agent. Subsequently, the surgical site was disinfected with Betadine. Using a dermatological pencil, a circle 0.5 cm in diameter was delineated on the back skin and incised using a scalpel and scissors to create a full-thickness excisional wound. Hydrogels were cut into circles with a diameter of 0.6 cm and a thickness of no more than 0.5 cm. As for PVA-P-FLL-E hydrogels, sterilization procedures were performed at two different stages. The first stage was to sterilize before the crosslinking of hydrogel. The PVA and pectin solution were first sterilized at 90 °C and then fabricated into a hydrogel under sterile conditions. The second stage was to perform terminal sterilization after the hydrogel had been crosslinked and was in its final form. All hydrogels were placed on sterilized tinfoil and exposed to UV irradiation for 30 min at a dose of approximately 250 nm in a class III/B3 biological safety cabinet (Thermo Fisher Scientific, Waltham, MA, USA) with a measured dose of 75 to 100 μW/cm^2^. Then, they were flipped using sterile tweezers and placed on new tinfoil for the second 30 min. The twenty-four male mice were randomly assigned to four groups (six mice per group): CWD (negative control), and PVA-P-FLL-E1, PVA-P, and FLL-E groups. The PVA-P-FLL-E1 and PVA-P hydrogels were applied every day until complete wound closure. The hydrogels were covered with a sterile gauze and secured using an adherent bandage. The FLL-E group received FLL-E treatment (similar dosage used for PVA-P-FLL-E1 production), while the CWD group remained untreated. The progression of wound closure was monitored through digital photography, and the wound diameter was measured at 1, 3, 6, 9, 11, and 15 days using a transparent ruler. The percentage of wound closure was calculated using Supplementary Equation S(4).

#### 3.8.3. Histopathological Studies

Tissue samples surrounding the scar or residual wound were collected post-euthanasia, fixed in 4% buffered formalin, dehydrated, and embedded in paraffin. Sections, each five microns thick, were prepared using a RM2245 rotary microtome (Leica, Wetzlar, Germany). These sections underwent staining using various methods: Hematoxylin and Eosin (H&E) to assess morphology, Masson’s Trichrome (MTC) to examine fibrosis and extracellular matrix (ECM) localization, and Picrosirius Red (PSR) to analyze collagen organization. Measurements of epidermal thickness and collagen area were conducted using computer-assisted image analysis based on the RGB (red, green, blue) system. Images of connective tissue were captured under polarized light (Axio Scope. A1, Zeiss, Oberkochen, Germany), and collagen fiber deposition was quantified in six random fields from these images. The total percentage of collagen fiber deposition was calculated using Image J 1.53e based on three different sections for each group.

#### 3.8.4. Immunohistochemistry (IHC) Evaluation

The sections were also subjected to IHC analysis to determine expressions of CK 14, CD45, CD34, CD 68 (Proteintech, Wuhan, China, NO. AP10143, 60287, 14486, 28058). Initial treatment of samples involved exposure to 0.5% hydrogen peroxidase/methanol and subsequent heat retrieval in 0.5 M citrate buffer (pH 6.0). Blocking Serum was applied for one hour at room temperature. The sections were then incubated overnight at 4 °C with primary antibodies: anti-CK14 (diluted in phosphate-buffered solution (PBS), 1:200), anti-CD45 (1:400), anti-CD34 (1:400), and anti-CD68 (1:400). Subsequently, they were incubated with a secondary antibody (horseradish peroxidase (HRP), Proteintech, No. SA00001) for 30 min at room temperature. Signal detection utilized diaminobenzidine (DAB, Proteintech, Wuhan, China, No. PK10002), and hematoxylin was used to counterstain the nuclei. Slides were mounted and digitally scanned at 10× magnification by five different observers, who individually counted 5 fields of view, and the average count was considered for each slide. Images were captured using an IX83 microscopy (Olympus, Tokyo, Japan), and positive staining colors were recorded and quantified automatically using Image J 1.53e software.

#### 3.8.5. Analysis of mRNA Expression by qRT-PCR

Skin tissue samples were processed using Trizol reagent (Invitrogen, Thermo Fisher Scientific, Inc.) for total RNA extraction. The purity and concentration of the extracted RNA in each sample were assessed by measuring the absorbance ratios at 260/280 nm and 260/230 nm using a NanoDrop 2000 ultraviolet–visible light spectrophotometer (Thermo, Waltham, MA, USA). The cDNA was synthesized using a StartScript first-strand cDNA synthesis Kit (GenStar, Co., San Francisco, CA, USA) as per the manufacturer’s instructions. qRT-PCR assays were performed on a 7500 Fast instrument (ABI, Inc., Tampa, FL, USA) using the RealStar Green Fast Kit (GenStar, Co., San Francisco, CA, USA). Primers targeting interleukin 1β (IL-1β), transforming growth factor-β (TGF-β), interleukin 10 (IL-10), tumor necrosis factor α (TNF-α), tissue inhibitor of metalloproteinase-1 (TIMP1), tissue inhibitor of metalloproteinase-2 (TIMP2), matrix metallopeptidase 9 (MMP9), matrix metallopeptidase 2 (MMP2), and glyceraldehyde-3-phosphate dehydrogenase (GAPDH) were acquired from Sangon Biotech Co., Ltd. (Shanghai, China). Each group involved five different individuals for extraction and analysis. Triplicate amplifications were performed for each sample, and the mean Ct value from technical replicates was utilized for final calculations. Supplementary Table S1 provides detailed information about each primer pair.

#### 3.8.6. Statistical Analysis

The statistical analysis was carried out using SPSS 11.0 statistical analysis software. Experimental outcomes were presented as mean ± standard deviation (SD) from three replicates. One-way analysis of variance (one-way ANOVA) was employed for result analysis with a significance level set at *p* < 0.05. To compare qualitative data in an organized manner, nonparametric test methods were utilized across the various groups.

## 4. Discussions

Wound healing is a fundamental response to tissue injury, prompting natural mechanisms that aim to repair damaged tissue and restore skin functionality [32]. It is imperative to use an appropriate dressing for the wound to prevent bacterial infection, and loss of body fluids, electrolytes, and nutrients, which may safeguard the quality of life and health of individuals [8]. An ideal wound dressing should create a moist environment around the wound while efficiently absorbing exudate from the wound surface. In this study, we developed cross-linked hydrogels using the freeze–thaw method, employing various combinations of PVA and pectin. Notably, the hydrogel possesses non-flow characteristics when applied to the wound and demonstrates coagulative abilities. Furthermore, the material, derived from PVA and pectin, exhibits a smooth surface along with excellent elasticity and tensile properties.

The FLL extract has exhibited remarkable antibacterial efficacy against various pathogens, as documented in prior studies [33]. These outcomes might be elucidated by the presence of triterpenoids and secoiridoids, compounds previously identified within the extract [34,35,36]. In this study, FLL-E demonstrated significant antibacterial activity, an observation that has been seldom reported in previous research. The enduring antibacterial effects observed in the PVA-P-FLL-E1 hydrogel against *S. aureus* and *E. coli* were attributed to the controlled release of FLL-E. The antibacterial effect of PVA-P-FLL-E1 hydrogel against both Gram-positive and Gram-negative bacterial strains demonstrates its potential for future application as a broad-spectrum antibiotic in wound healing.

Beyond conferring anti-infective properties to skin dressings, enhancing their structural and swelling characteristics is equally crucial [37,38,39]. As shown by SEM, PVA-P-FLL-E hydrogels had small and irregular micropores, indicating advantages in preventing microbial infection around wound sites [40]. In this work, the addition of FLL-E enhanced the hydrophobic property, increased porosity and adhesive strength, and avoided the deformation and breakage inside the hydrogel. PVA-P-FLL-E hydrogels were less swellable compared to PVA-P itself because of their higher hydrogen bond formation between hydroxyl groups, preventing extensive shrinkage of the network structure and tightly holding water molecules. Consequently, water release was mitigated. This is supported by the reduction in hydroxyl group signals of PVA-P-FLL-E1 and PVA-P-FLL-E2 hydrogels as compared to FLL-E, pectin, and PVA in the FT-IR absorption spectra in Figure 1j. 

The physical crosslinks play a dominant role in the performance improvement of PVA-P-FLL-E hydrogel. XRD results in Figure 1k showed that the addition of pectin into PVA resulted in a drop in peak intensity of PVA-P, indicating that the pectin is able to disrupt crystalline domains and decrease overall crystallinity. This result suggests that crosslinking interactions occurred: crystallization in PVA due to hydrogen bonding, and interaction between PVA and hydroxyl groups present in the backbone of pectin. The crosslinking between PVA and pectin results in an interpenetrating polymer network structure [41,42]. In addition, the introduction of FLL-E into PVA-P did not cause a marked decrease in peak intensity of PVA-P-FLL-E, indicating that FLL-E likely had minimal interference in the crystallization process. Since there are more hydroxyl groups on both PVA-P and FLL-E, the hydrogen bond between PVA-P and FLL-E may also be formed. The synergistic combination of different crosslinking mechanisms in an interpenetrating polymer network structure results in remarkably improved water retention capacity properties for PVA-P-FLL-E hydrogels. 

Similar to the water retention capacity characteristic, PVA-P-FLL-E hydrogels also exhibited a continuous-release effect of FLL-E throughout the entire hydrogel network within 24 h. Local application of PVA-P-FLL-E hydrogel can provide high concentrations of FLL-E at wound sites, offering sustained protection during the wound healing process and circumventing systemic effects. However, PVA-P-FLL-E hydrogels are not suitable for long-term drug delivery, because of the uncontrolled diffusion of drugs from the swollen hydrogels. Our results implied that the hydrogels needed to be replaced every 24 h. Notably, compared to PVA-P-FLL-E2 hydrogel, the adhesive strength for PVA-P-FLL-E1 hydrogel was less, so it will not cause a secondary injury once it has been removed from wound surfaces; the PVA-P-FLL-E1 hydrogel displayed minimal chromatic aberration, high drug storage capacity, and superior hydrogel-forming characteristics.

The application of PVA-P-FLL-E1 hydrogel exhibited no immediate adverse effects on the skin and demonstrated superior wound healing potential compared to both PVA-P hydrogel and CWD. This enhanced healing capability is attributed to the potential therapeutic effect of FLL-E. PVA-P-FLL-E1 hydrogel not only maintained optimal moisture levels but also effectively managed wound exudates, thereby supporting efficient healing while displaying remarkable elasticity, compared to the CWD. Besides analyzing the effect of PVA-P-FLL-E in promoting wound healing, this study also analyzed its mechanisms. There are several steps involved in wound healing, including inflammation, cell migration and proliferation, angiogenesis, neovascularization, production of extracellular matrix (ECM), and remodeling [43]. Central to ECM formation is collagen, particularly types I and III (less so types IV, V, VI, and VIII), pivotal for dermal tissue reconstruction at wound sites [44,45]. Collagen composition is critical to maintaining density and mechanical strength in regenerates. Healing wounds resulted in changes in collagen distribution and remodeling of the ECM, and the changing ratio of collagen I to collagen III during wound healing is crucial. A healing wound is associated with increased collagen III, while a healed wound is associated with increased collagen I [46]. Here, to promote collagen deposition, PVA-P-FLL-E1 hydrogel was engineered to serve as an ECM-mimicking dressing for bacteria-infected cutaneous wound healing without additional bactericidal properties, cytokines, or cells. Accordingly, the PVA-P-FLL-E1 treatment promoted collagen I deposition and showed heightened expression of collagen I compared to the three other groups, indicating its potential to promote wound healing. Structurally, pectin forms a strong physical bond with the surface glycocalyx of mammalian visceral organs, making it similar to the extracellular surface layer of mammalian cells [47,48]. This property facilitates cell adhesion. Thereby, it can provide a structural scaffold for wound healing [49]. Notably, PVA is mechanically advantageous for collagen deposition despite lacking cell adhesion capability [50]. Moreover, to combat the infection at the wound site, a natural product of FLL-E was used, reducing the pathogenic bacteria and stabilizing the microenvironment around the wound area. A better understanding of such fundamental mechanisms is that the PVA-P-FLL-E1 hydrogel was designed to facilitate ECM formation by effectively providing an optimal structure and functional microenvironment.

Furthermore, the modulation of collagen I and collagen III levels is influenced by differential gene expression and degradation, often regulated by enzymes like metalloproteinases (MMPs) [46]. Among the MMPs, MMP2 and MMP9 are two of the most important, and their downregulation may improve the quality of wound healing [51]. Simultaneously, increased expression of tissue inhibitors of metalloproteinases (TIMPs), such as TIMP1 and TIMP2, acts as a natural inhibitor of MMP2 and MMP9, facilitating improved wound healing by shortening the skin regeneration period [52]. In line with previous research, the study demonstrated that PVA-P-FLL-E1 hydrogel promoted wound healing by downregulating MMP2 and MMP9 mRNA expressions while upregulating TIMP1 and TIMP2 mRNA levels, corroborating its efficacy in fostering efficient wound closure and healing.

At every stage of healing, CD68- and CD45-positive cells were present in the dermis, indicating an inflammatory response [53]. CD34 has a pivotal role in regulating postnatal physiological and pathological angiogenesis [54]. Neovascularization is essential for the re-epithelialization process, due to the fact that it can ensure adequate nutrient and oxygen supply for the migration and proliferation of epidermal and dermal cells. The cytokeratin 14 (CK14) marker is crucial in evaluating epidermal differentiation and wound healing [55]. Lower levels of CD45 and CD68 were detected at the wound sites of the PVA-P-FLL-E1 group compared with the other three groups, indicating that PVA-P-FLL-E1 reduced the immune response at the wound sites. Higher expressions of CD34 and CK14 during keratinocyte differentiation were observed at the wound surfaces of PVA-P-FLL-E1-treated animals, suggesting that the topical application of PVA-P-FLL-E1 hydrogel to wounds appears to have the potential to hasten the migration of keratinocytes towards the injured area and stimulate increased keratinocyte proliferation in the basal layer, ultimately leading to the formation of a thicker neoepidermis. The inflammatory response is also fundamental to wound healing, since it aids in hemostasis, attacks invading pathogens, and removes debris from the wound. While inflammation is crucial for wound healing, its prolonged or excessive presence can lead to delayed healing. In this study, the application of PVA-P-FLL-E1 hydrogel appears to expedite the inflammatory phase, aiding in reducing wound area.

## 5. Conclusions

The incorporation of pectin in the fabrication of PVA-P hydrogel enhanced the ability of the PVA-P hydrogel to adhere to tissues. The addition of FLL-E resulted in an enhanced hydrogel optimal for wound healing, exhibiting statistically significant improvements in antibacterial properties compared to PVA-P hydrogel. The enhanced functionality and physicochemical properties may depend on the incorporation of pectin and FLL-E into the PVA crystalline structure, thereby increasing the number of physical hydrogen bonds. PVA-P-FLL-E1 hydrogel promoted wound closure by enhancing collagen synthesis, preventing dysfunction in ECM remodeling via TIMP signaling, accelerating the re-epithelialization process, degrading various inflammatory factors, and enhancing cell proliferation. This study provides evidence of the effectiveness of utilizing PVA-P-FLL-E1 hydrogel in wound treatment, reveals the potential mechanism of PVA-P-FLL-E1 hydrogel-accelerated wound healing, and highlights a therapeutic strategy involving in the use of PVA-P-FLL-E1 hydrogel for wound repair. However, the clinical value of these hydrogels in tissue engineering should be further evaluated.

## Data Availability

The data presented in this study are available on request from the corresponding author.

## Supplementary Materials

The following supporting information can be downloaded at: https://www.mdpi.com/article/10.3390/molecules29071423/s1, Figure S1: The HPLC, ^1^H NMR and ^13^C NMR spectra of FLL-E; Figure S2:The characterization of PVA-P hydrogels with different mass ratios of PVA and pectin were analyzed by scanning electron microscopy (SEM); Figure S3: Porosity comparison of PVA-P hydrogels with different mass ratios of PVA and pectin; Figure S4: Contact angle of PVA-P hydrogels with different mass ratios of PVA and pectin; Figure S5: The UV, FT-IR and XRD spectra of PVA-P hydrogels with different mass ratios of PVA and pectin; Figure S6: Statistical analysis results of swelling ratio, water retention ratio and degradation from PVA-P hydrogels with different mass ratios of PVA and pectin; Figure S7: Adhesive strength, thickness and color measurement comparison of PVA-P hydrogels with different mass ratios of PVA and pectin; Figure S8: Porosity, contact angels and UV spectra comparison of PVA-P-FLL-E1, PVA-P-FLL-E2 and PVA-P hydrogels; Figure S9: Color measurement and adhesive strength comparison of PVA-P-FLL-E1, PVA-P-FLL-E2 and PVA-P hydrogels; Table S1: Primer sequence and amplicon lengths of every assay; Figure S10: In vivo biocompatibility assessment of FLL-E; Equation S(1): Porosity calculation formula; Equation S(2): Swelling ratio (SR), water retention ratio (WR), and degradation ratio (DR) calculation formula; Equation S(3): ZOI calculation formula; Equation S(4): Wound closure calculation formula.

## Abbreviations

PVA: polyvinyl alcohol; FLL-E: *Fructus Ligustri Lucidi* polysaccharides; PVA-P-FLL-E1: polyvinyl alcohol/pectin with FLL-E1; PVA-P-FLL-E2: polyvinyl alcohol/pectin with FLL-E2; PVA-P: polyvinyl alcohol/pectin; DEAE: diethylaminoethyl; HPLC: High performance liquid chromatography; NMR: nuclear magnetic resonance; SEM: Scanning electron microscope; SR: swelling ratio; WR: water retention ratio; DR: degradation ratio; FT-IR: fourier-transform infrared; XRD: X-Ray diffractometry; UV: ultraviolet; *S. aureus*: *Staphylococcus aureus*; *E. coli*: *Escherichia coli*; CWD: negative control; MTC: Masson’s trichrome; PSR: Picrosirius Red; CK14: Cytokeratin 14; IL-1β: interleukin 1β; TGF-β: transforming growth factor-β; IL-10: interleukin 10; TNF-α: tumor necrosis factor α; TIMP1: tissue inhibitor of metalloproteinase-1; TIMP2: tissue inhibitor of metalloproteinase-1; MMP9: matrix metallopeptidase 9; MMP2: matrix metallopeptidase2; ECM: extracellular matrix; Man: mannose; Glc N: glucosamine; Rib: ribose; Rham: rhamnose; GlcUA: glucuronic acid; GalUA: galacturonic acid; Glc: glucose; Gal: galactose; Xyl: xylose; Ara: arabinose; Fuc: fucose.
