# Supplementary material for: Antibacterial and Anti-Inflammatory Polysaccharide from Fructus Ligustri Lucidi Incorporated in PVA/Pectin Hydrogels Accelerate Wound Healing
Source: Molecules. 2024 Mar 22;29(7):1423. doi: 10.3390/molecules29071423 (PMC11012603; doi:10.3390/molecules29071423)
Supplement: Supplementary file 1 [file molecules-29-01423-s001.zip › molecules-2854247-supplementary.pdf]

Supplementary Information for the publication

Antibacterial and Anti-Inflammatory Polysaccharide from *Fructus Ligustri Lucidi* Incorporated in PVA/Pectin Hydrogels Accelerate Wound Healing

Yanli Xi [1], Lianxin Hu [2], Xiang Chen [1], Lili Zuo [3], Xuesong Bai [4], Weijie Du [1] and Na Xu [5,*]

1 Department of Toxicology, School of Public Health, Jilin Medical University, Jilin 132013, China; jilin2534126@163.com (Y.X.); 18755818472@163.com (X.C.); 17835600176@163.com (W.D.)
2 Department of Clinical Medicine, School of Clinical Medicine, Jilin Medical University, Jilin 132013, China; 13634445675@163.com
3 Department of Food Quality and Safety, School of Public Health, Jilin Medical University, Jilin 132013, China; zuolili213@163.com
4 Department of Nutrition, School of Public Health, Jilin Medical University, Jilin 132013, China; baixueso@163.com
5 Office of Educational Administration, Jilin Medical University, Jilin 132013, China
* Correspondence: xunajlu@sina.com

1.1 FLL-E extraction and purification

Fructus Ligustri Lucidi (FLL, the dried mature fruit of *Ligustrum lucidum* Ait.) was purchased from Beijing Tongrentang Pharmaceutical Co. Ltd., China. The herb was washed with tap water, air dried, and powdered. Authentication and standardization of the herb were conducted meticulously, adhering to the criteria outlined in the Chinese Pharmacopoeia (2020), with a focus on its marker compounds. The processed herb was stored in a dry, sealed container at 4°C to prevent moisture absorption and preserve its quality.

The active ingredients of FLL were prepared through a modified hot water extraction process based on established methods.[1] Approximately 10 grams of FLL powder underwent extraction three times using 100 mL of hot water for durations of 3 h, 2 h, and 1 h at 60°C within a reflux apparatus. The resulting extracts were combined, filtered, and concentrated under reduced pressure until reaching a volume of about 30 mL. These extracts were then precipitated overnight at 4°C using four volumes of 95% ethanol (v/v). The precipitates were filtered, washed, and concentrated until no alcohol precipitation was observed. The resulting precipitates were dissolved in distilled water, treated with Sevag's solution (1/3 volume chloroform/n-butanol (4:1, v/v)), and agitated rigorously to remove proteins. Subsequently, three rounds of petroleum ether extraction were conducted to eliminate pigments. The extracts were washed with DEAE cellulose until achieving a colorless state, eluted with 0.1 mol/L NaCl, dialyzed against a membrane with a molecular weight cut-off (MWCO) of 0.5 kDa for 60 h to remove salt,

and then freeze-dried to obtain a yellow powder. The yield of FLL-E was determined using the phenol-sulfate method.[2] The resulting FLL extracts were dissolved in distilled water at appropriate concentrations.

1.2 Monosaccharides and molecular weight analysis of FLL-E

High-performance liquid chromatography (HPLC) using an Agilent 1100 system (USA) was employed for compound analysis. An Agilent Zorbax SB-C18 column (4.6×250 mm, 2.7 μm) maintained at 30°C was utilized for compounds analysis, with the mobile phase comprising 18% acetonitrile buffer at a flow rate of 0.8 mL/min. Detection of compounds occurred via a UV detector at 245 nm. Prior to injection, samples were dissolved in 18% acetonitrile buffer to achieve a final concentration of 10 mg/mL, and the sample solution was filtered through a 0.45 μm filter membrane. Each run involved the injection of 20 μL of the sample. Additionally, molecular weight calculations were performed using the Agilent 1100 HPLC system (USA). Analyst Software GPC facilitated data processing. An Agilent Ultrahydrogel™ Linear column (300 mm×7.8 mm(id)×2) was maintained at 45°C, with a mobile phase consisting of 0.1 mol/L sodium nitrate at a flow rate of 0.9 mL/min, and detection accomplished using a refractive index detector. The estimation of molecular weight was based on a calibration curve derived from known molecular weight standards (T-2000, T-150, T-40, T-10, and T-5) of the Dextran T-series.

1.3 Assessment results of FLL-E

Based on the phytochemical test (*Pharmacopoeia of the People's Republic of China*, 2020 Edition), the polysaccharide content of the extracts was about 755.10±44.88 mg/g (calculated by FLL-E). HPLC analysis demonstrated that the FLL-E consists of 11 monosaccharides, namely, mannose (Man), glucosamine (GlcN), ribose (Rib), rhamnose (Rham), glucuronic acid (GlcUA), galacturonic acid (GalUA), glucose (Glc), galactose (Gal), xylose (Xyl), arabinose (Ara), fucose (Fuc) in a molar ratio of 3.80:0.25:0.58:5.95:1.13:27.28:23.06:12.31:2.03:23.20:0.41 (Figure S1a), and the molecular weight of the FLL-E were 115156 Da (Figure S1b).

1.4 NMR spectral analysis of FLL-E

The NMR spectra of FLL-E was obtained by Avance-500 NMR spectrometer (Bruker Inc., Rheinstetten, Germany). All compounds were dissolved in D_2O. The ^1H and ^{13}C NMR spectra of FLL-E were recorded.

1.5 Results of NMR spectral analysis

The ^1H NMR spectrum (Figure S1c) displayed a signal at 4.75 ppm, corresponding to the D_2O. Additionally, a signal peak around 2 ppm was attributed to CH_3-COO. In the ^{13}C NMR spectrum (Figure S1d), FLL-E exhibited signals in the low field (160 to 180 ppm), indicating the presence of uronic acid. Specifically, the CHOH and CH_2OH peaks were observed at 54.98 ppm, while carbohydrate residues appeared around 20 ppm. These NMR findings suggested that FLL-E comprised acidic polysaccharides, aligning with the monosaccharide composition.

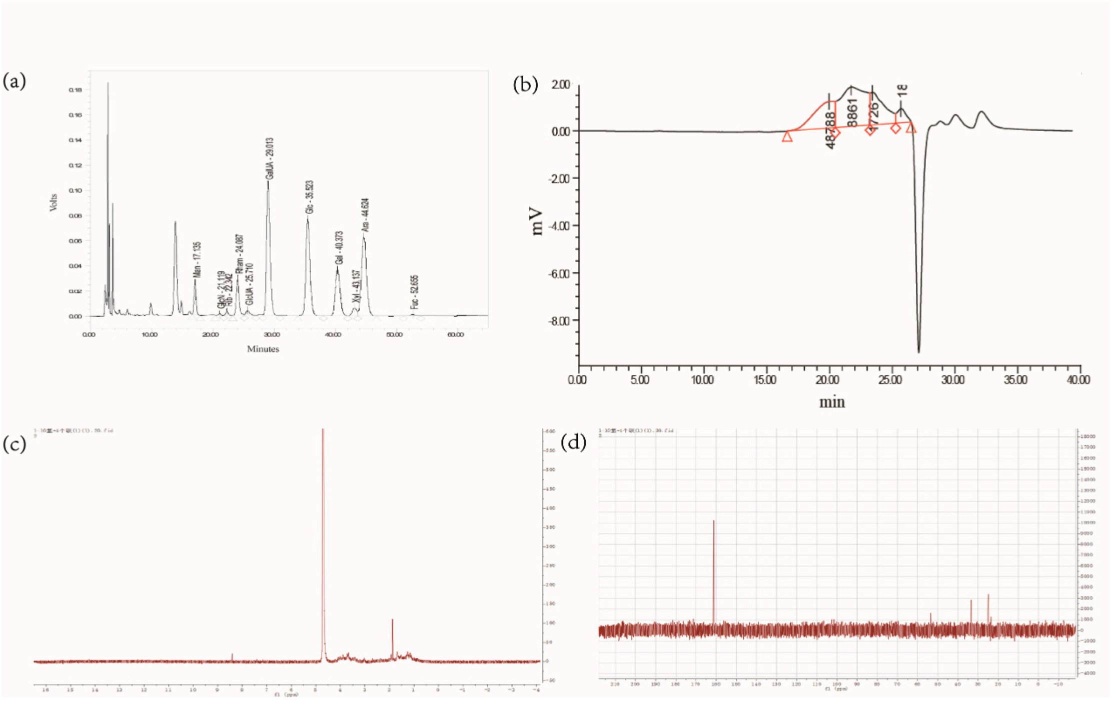

Figure S1. The HPLC(**a**,**b**), ^{1}H NMR(**c**) and ^{13}C NMR(**d**) spectra of FLL-E.

1.6 Characterizations of PVA-P hydrogels

To promote superficial wound repair as much as possible, the network structure and physical properties of PVA-P hydrogels should be optimized. Thus, PVA-P hydrogels with different weigh ratios of PVA to pectin (2:8,4:6,5:5,6:4,8:2) were prepared. First, the SEM analysis (Figure S2a-e) revealed distinct 3D porous structures across all samples, showcasing irregular interconnected areas. As the pectin percentage increased within the PVA-P hydrogels, both the network's porosity and pore sizes expanded. This phenomenon is known to facilitate accelerated drug release from within the hydrogel structure.[3] Among these structures, the surface morphologies of PVA: pectin (6:4) and PVA: pectin (8:2) hydrogels had higher homogeneous appearance of the pores, and this is better for the homogeneous drug loading of the entire PVA/pectin polymer network. The porosity rate of PVA: pectin (6:4) was greater than 50% that allowed the diffusion of water molecules into hydrogel resulting in swelling property (Figure S3).

Although the addition of PVA to the hydrogels resulted in an improvement in the hydrophobicity, the exception was PVA: pectin (8:2) (Figure S4a-f). Previous studies revealed the hydrophobic surface of the material is more conducive to protein and cell adhesion.[4,5] The contact angel value of PVA: pectin (6:4) was around 36.11°, which is most conductive to cell adhesion of all samples.

The chemical structure of PVA: pectin was investigated by UV, FT-IR and XRD. The examination of PVA:pectin's chemical structure through UV, FT-IR, and XRD analyses showed no significant differences across varying ratios of PVA-P hydrogels (Figure S5a-c). Notably, the FT-IR peak at 3440 cm^{-1}, attributed to the hydroxyl group, appeared consistent among the different hydrogel compositions (Figure S5b). Through heat treatment, the PVA chains exhibited enhanced crystalline structures, leading to reduced

free hydroxyl groups and indicating increased crystallinity. The XRD pattern of PVA:pectin showcased typical crystalline states, signifying greater hydrogen bonding formation between PVA and pectin chains.

FigS6a demonstrated that all hydrogels displayed rapid moisture absorption and expansion. The observed reduction in swelling ratio (SR) from 2:8 to 8:2 corresponded to the increased proportion of PVA within the hydrogels. A higher PVA content resulted in a more rigid and denser network structure, leading to decreased water absorption due to lower contractility. This change can be attributed to the formation of hydrogen bonding between PVA chains, consistent with findings from SEM analyses.

Furthermore, with increased pectin concentration, the hydrogels exhibited higher water retention rates (FigS6b). This improvement in water retention capacity due to pectin incorporation aligns with previous findings where sucrose-enhanced PVA hydrogels displayed reduced swelling attributed to increased hydrogen bonding and chain density.[6] The presence of water-soluble pectin molecules in the network likely facilitated enhanced water penetration into the hydrogel.

Interestingly, the PVA:pectin (2:8) hydrogel showed the fastest degradation rate (Figure S6c). This outcome suggests that excessive pectin content facilitated easier degradation of the PVA-P hydrogel. This excessive introduction of pectin might create a less compact structure, compromising hydrogel stability and leading to easier degradation. Hence, meticulous control over the PVA and pectin concentration ratio is critical for crafting robust multifunctional composite hydrogels.

PVA: pectin (8:2) hydrogel showed the best adhesive strengths, and its thickness was also the highest (Figure S7a and b). A diminishing ΔE (ranging from 2:8 to 6:4) with increasing PVA concentration was observed for the hydrogels, exception for the PVA: pectin (8:2) hydrogel (Figure S7c). The ΔE was the highest for PVA: pectin (8:2) hydrogel.

High adhesion may cause secondary damage when changing wound dressings. Thicker wound dressings tend to detach from the wound site when applied. Besides, the appearance of PVA: pectin (8:2) hydrogel was hard and rubbery. The adhesive strength and thickness of PVA: pectin (6:4) hydrogel were inferior to that of PVA: pectin (8:2) hydrogel, but the appearance of it was elastic.

Consequently, the mass ratio of PVA to pectin was fixed at 6:4 (w/w) for subsequent studies.

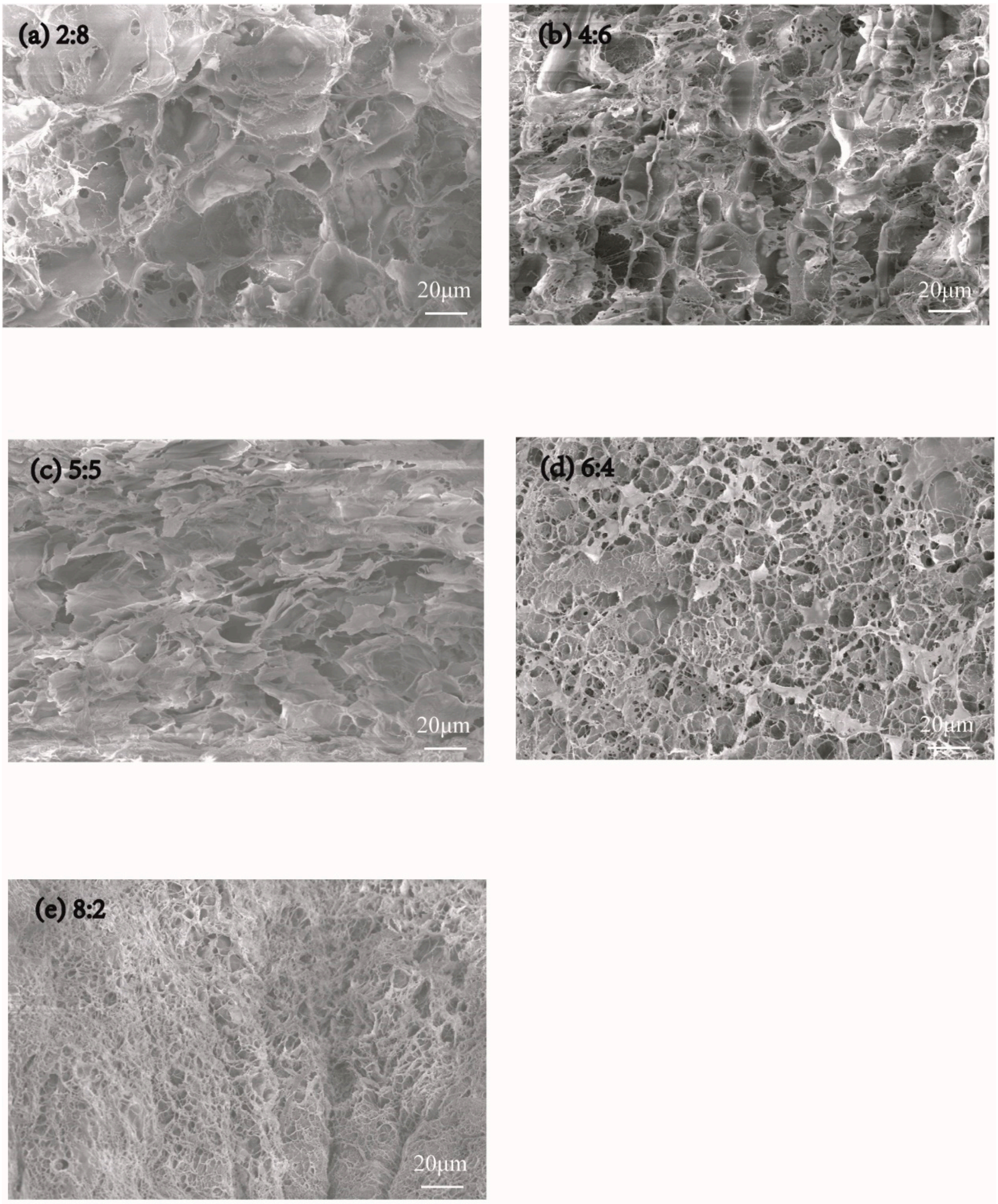

Figure S2. The characterization of PVA-P hydrogels with different mass ratios of PVA and pectin were analyzed by scanning electron microscopy (SEM). SEM scale bars: 20 μm. SEM magnification: ×500.

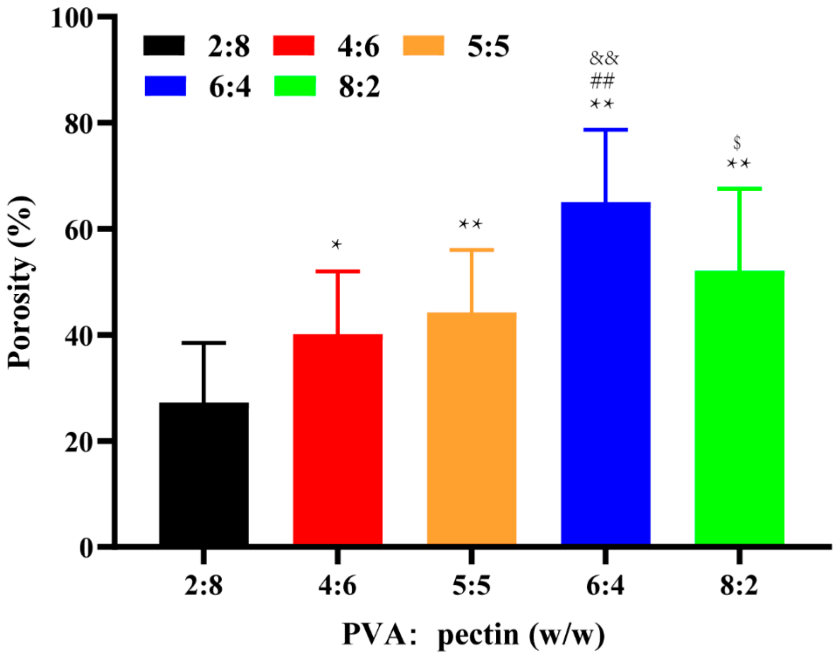

Figure S3. Porosity comparison of PVA-P hydrogels with different mass ratios of PVA and pectin. Error bars indicate SD. Significant differences between sample means are indicated. $^{*}p<0.05$, $^{**}p<0.01$ versus PVA-P (2:8) hydrogel; $^{\#\#}p<0.01$ versus PVA-P (4:6) hydrogel; $^{\&\&}p<0.01$ versus PVA-P (5:5) hydrogel; $^{\$}p<0.05$ versus PVA-P (6:4) hydrogel.

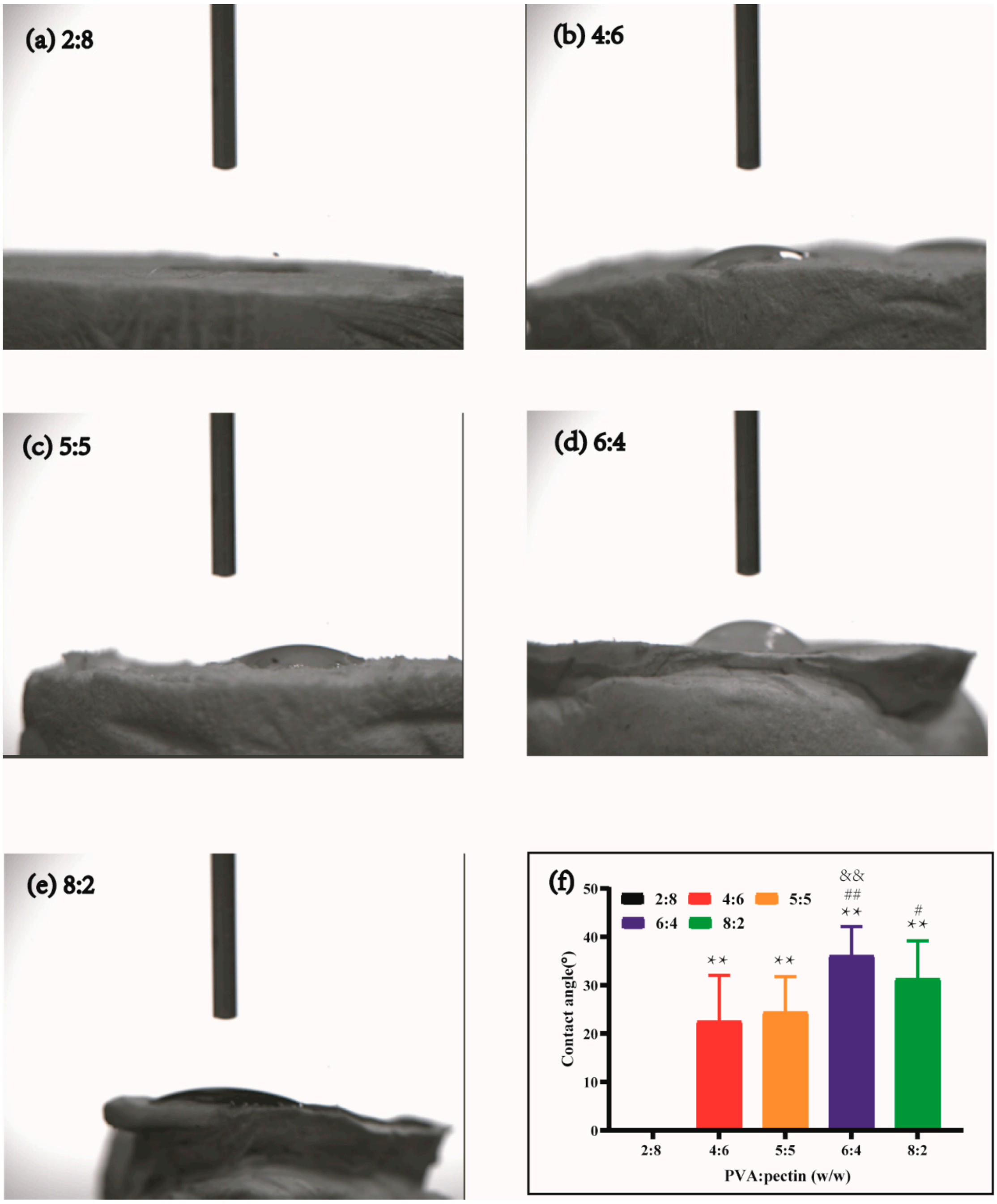

Figure S4. Contact angle of PVA-P hydrogels with different mass ratios of PVA and pectin (**a**–**e**). Statistical analysis results of average contact angle from PVA-P hydrogels were shown in (**f**). Error bars indicate SD. Significant differences between sample means are indicated; $^{**}p<0.01$ versus PVA-P (2:8) hydrogel; $^{\#}p<0.05$, $^{\#\#}p<0.01$ versus PVA-P (4:6) hydrogel; $^{\&\&}p<0.01$ versus PVA-P (5:5) hydrogel.

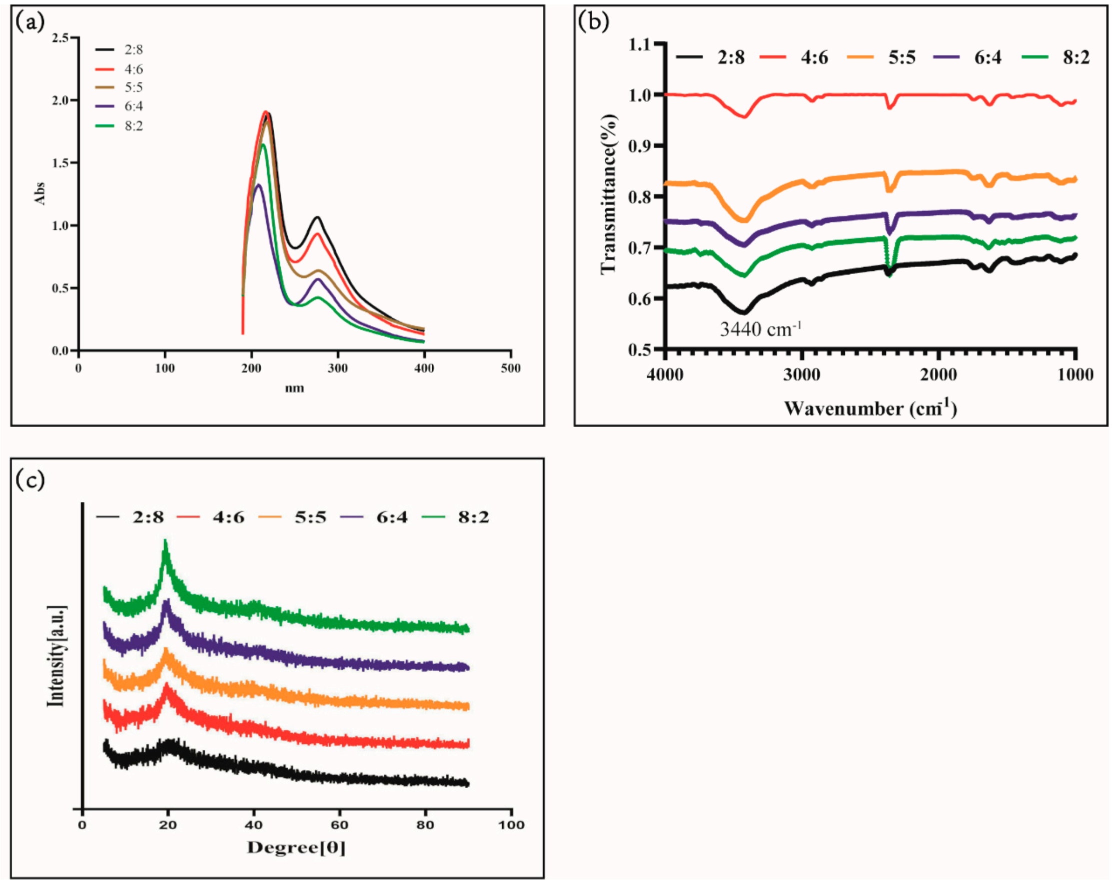

Figure S5. The UV(**a**), FT-IR(**b**) and XRD(**c**) spectra of PVA-P hydrogels with different mass ratios of PVA and pectin.

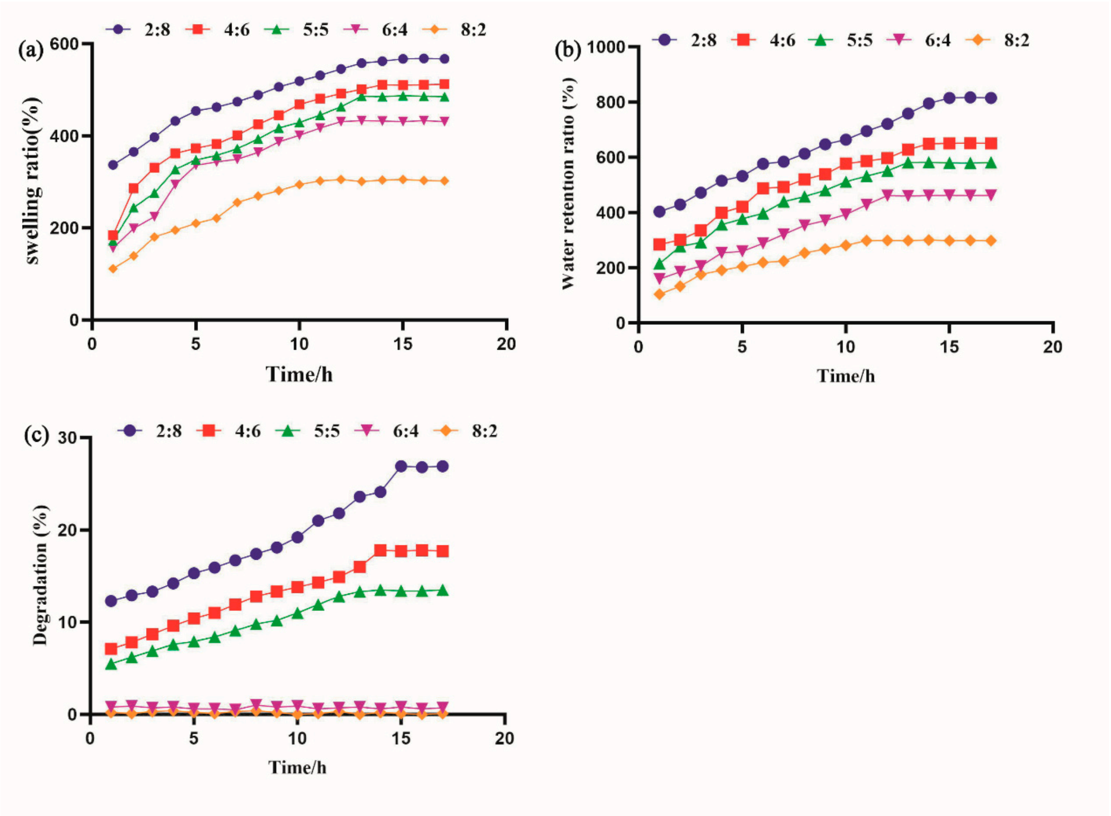

Figure S6. Statistical analysis results of swelling ratio (SR, **a**), water retention ratio (WR, **b**) and degradation (DR, **c**) from PVA-P hydrogels with different mass ratios of PVA and pectin.

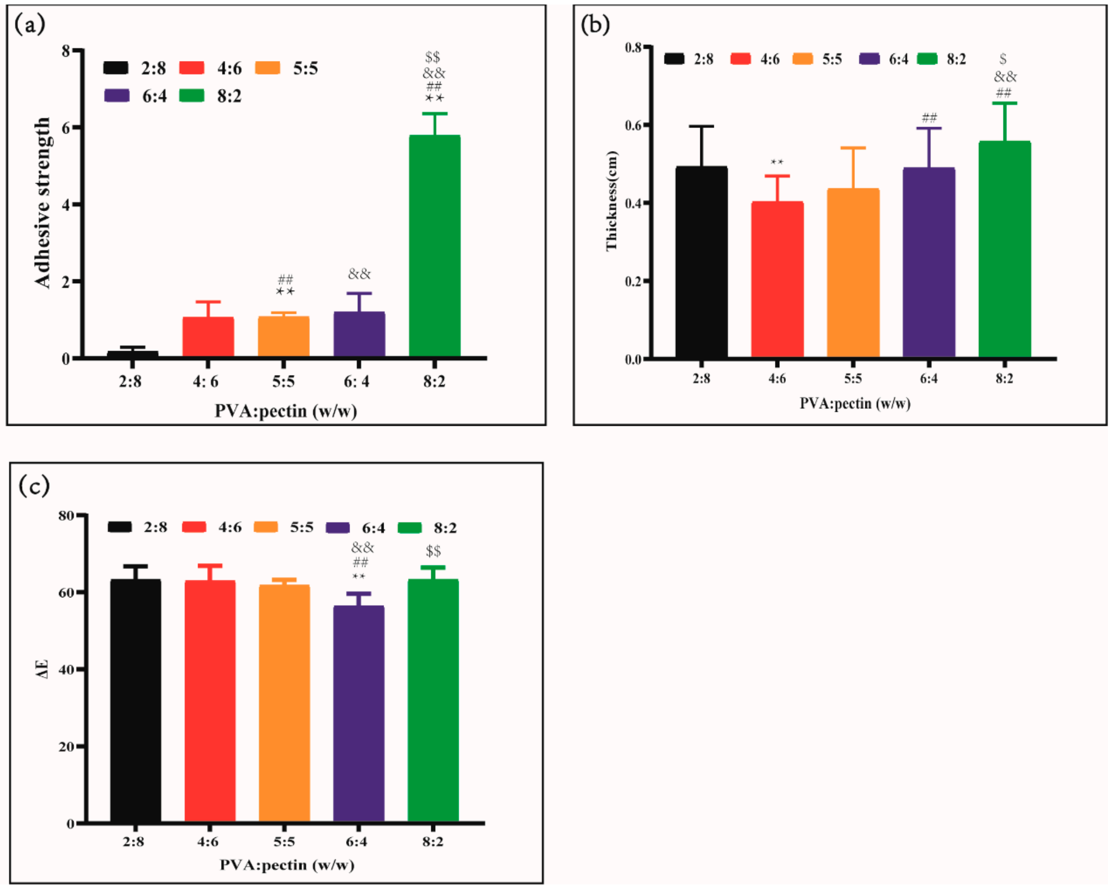

Figure S7. Adhesive strength (**a**), thickness(**b**) and color measurement (ΔE, **c**) comparison of PVA-P hydrogels with different mass ratios of PVA and pectin. Error bars indicate SD. Significant differences between sample means are indicated. $^{**}p<0.01$ versus PVA-P (2:8) hydrogel; $^{\#\#}p<0.01$ versus PVA-P (4:6) hydrogel; $^{\&\&}p<0.01$ versus PVA-P (5:5) hydrogel; $^{\$}p<0.05$, $^{\$\$}p<0.01$ versus PVA-P (6:4) hydrogel.

1.7 Porosity, contact angels and UV spectra comparison of PVA-P-FLL-E1, PVA-P-FLL-E2 and PVA-P hydrogels

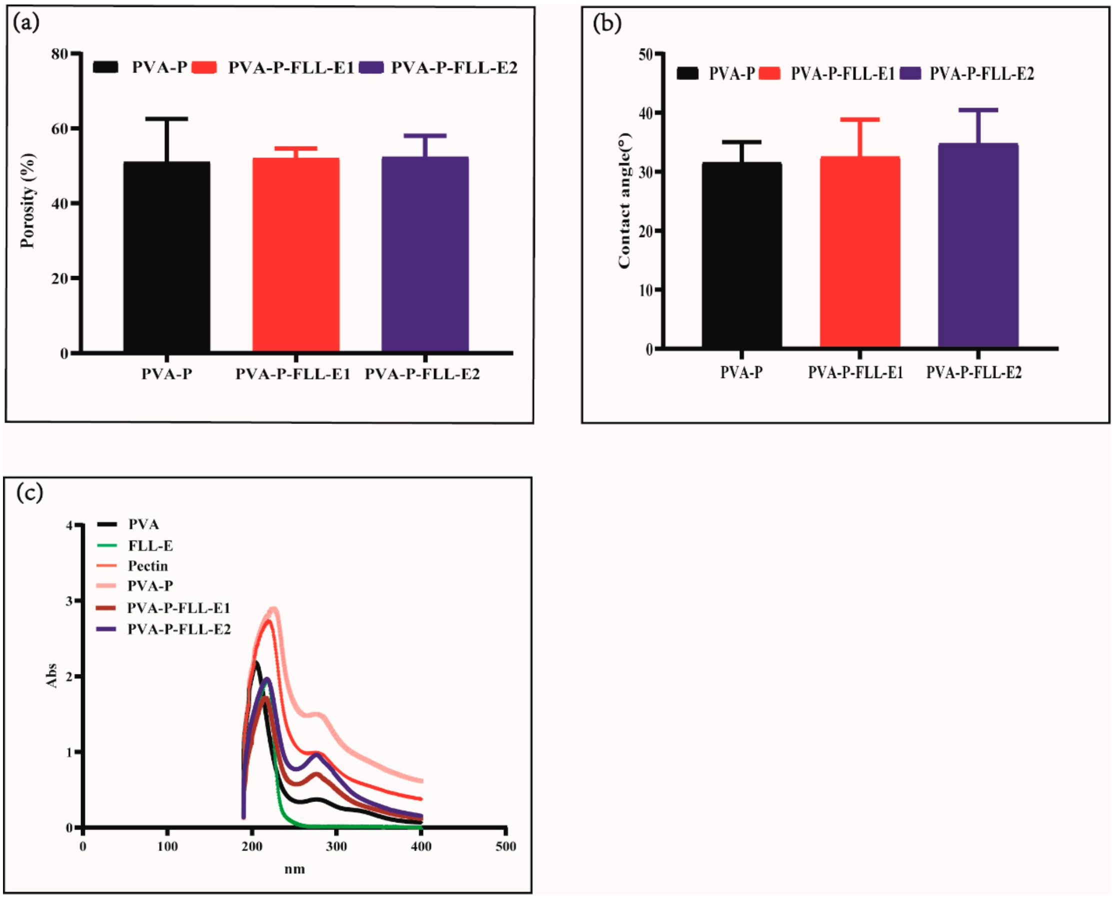

Figure S8. Porosity (**a**), contact angels(**b**) and UV spectra(**c**) comparison of PVA-P-FLL-E1, PVA-P-FLL-E2 and PVA-P hydrogels. Error bars indicate SD.

1.8 Color measurement and adhesive strength comparison of PVA-P-FLL-E1, PVA-P-FLL-E2 and PVA-P hydrogels

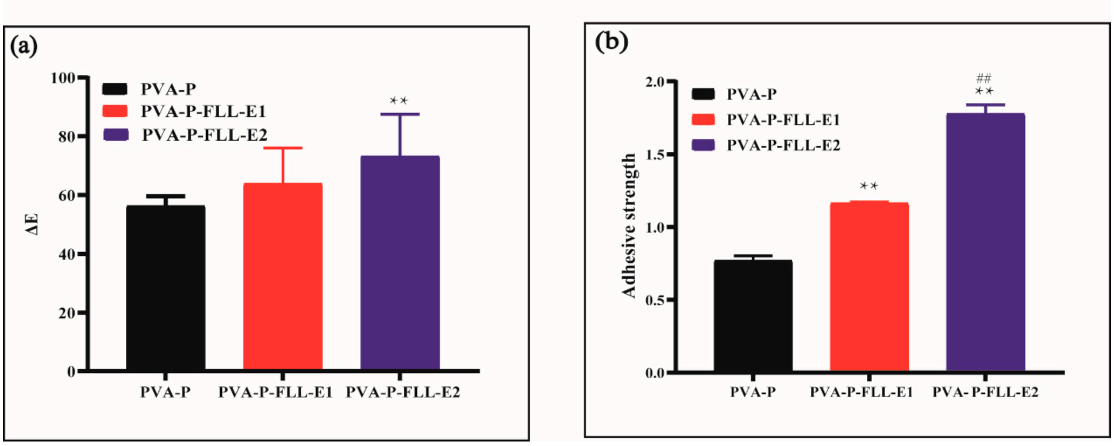

Figure S9. Color measurement (ΔE, **a**) and adhesive strength (**b**) comparison of PVA-P-FLL-E1, PVA-P-FLL-E2 and PVA-P hydrogels. Error bars indicate SD. Significant differences between sample means are indicated. $^{**}p<0.01$ versus PVA-P hydrogel; $^{\#\#}p<0.01$ versus PVA-P-FLL-E1 hydrogel.

1.9 Primer sequence

Table S1. Primer sequence and amplicon lengths of every assay.

Gene name	Primer sequence (5'→3')	Accession number	Amplicon length(bp)	Tm	%GC
IL-1*β*	AGAAGCTGTGGCAGCT	NM_008361.4	515	55.20	56.25
	TGAGGTGCTGATGTACCA			55.03	50.00
IL-10	GCTCTTACTGACTGGCATGAG	NM_010548.2	105	58.45	52.38
	CGCAGCTCTAGGAGCATGTG			60.88	60.00
MMP2	ACCACCTTAACTGTTGCTTTTG	NM_008610.3	79	57.61	40.91
	AGGAAATGCAGTGGAGTGGAA			59.57	47.62
MMP9	GGACGACGTGGGCTACGT	NM_013599.5	75	61.74	66.67
	CACGGTTGAAGCAAAGAAGGA			59.05	47.62
TGF-β	TAGGAAGGACCTGGGTTGGAA G	NM_011577.2	139	61.10	54.55
	CGGGTTGTGTTGGTTGTAGAGG			61.38	54.55
TNF-α	GAACTGGCAGAAGAGGCACT	NM_013693.3	201	59.96	55.00
	GGTCTGGGCCATAGAACTGA			58.80	55.00
TIMP1	CCTTCGCATGGACATTATTCTC	NM_011593.2	132	57.03	50.00
	TCTCTAGGAGCCCCGATCTG			58.57	50.00
TIMP2	TACCGGTTCTGAAAGACGGC	NM_011594.3	102	60.04	55.00
	CCCAAAGGTTCGTTTGCTCG			60.04	55.00
GAPDH	GGTCCCAGCTTAGGTTCATCA	NM_001289726.2	83	59.44	52.38
	CCGTTCACACCGACCTTCA			59.93	57.89

Abbreviate: IL-1β: interleukin 1β; TGF-β: transforming growth factor-β; IL-10: interleukin 10; TNF-α: tumor necrosis factor α; TIMP1: tissue inhibitor of metalloproteinase-1; TIMP2: tissue inhibitor of metalloproteinase-1; MMP9: matrix metallopeptidase 9; MMP2: matrix metallopeptidase2 and GAPDH: glyceraldehyde-3-phosphate dehydrogenase; Tm: melting temperature.

1.10 Equation

Equation S1

The porosity of the hydrogels was determined via the solvent displacement method. Initially, the hydrogels underwent freeze-drying and were weighed (Wd). Subsequently, these freeze-dried samples were saturated by immersing them in absolute ethanol and reweighed (Wf), while measuring the volume of ethanol absorbed (Vs). The porosity percentage was calculated using the equation (1):

$$\text{Porosity /\%}=\frac{(\mathrm{Wf}-\mathrm{Wd})}{\mathrm{Vs}\times\rho\mathrm{ethanol}}\times 100 \qquad (1)$$

Equation S2

The swelling ratio (SR) of samples was calculated based on equation (2); the water retention ratio (WR) was determined by equation (3); the degradation ratio (DR) was calculated based on equation (4).

Samples (5×5 cm^2) were freeze-dried and kept in desiccator until the measurement(recorded as Wd). Then the samples were swollen in PBS (pH=7.4) at 37°C for 24 h and wrapped with filter paper to remove excess water for the weight measurement (recorded as Ws). They were used to analyzed the SR.

$$\text{SR/\%}=\frac{(\mathrm{Ws}-\mathrm{Wd})}{\mathrm{Wd}}\times 100 \qquad (2)$$

The dried samples were dipped into PBS at 37°C for 48 h and wrapped with filter paper to remove excess water. Then the samples were transferred to beaker. Samples were weighted at a preset time and recorded as Wr. Finally, the samples were dried at 60°C to constant weight and recorded as Wc. Wr and Wc were used to analyzed the WR.

$$\mathrm{WR}(\%)=\frac{\mathrm{Wr}-\mathrm{Wc}}{\mathrm{Wc}}\times 100 \qquad (3)$$

The dried samples were weighted, recorded as Wi and dipped into PBS at 37°C. Then samples were taken out and wrapped with filter paper to remove excess water at a preset time. Finally, the samples were dried at 60°C to constant weight and recorded as Wd. They were used to analyzed the DR.

$$\mathrm{DR}(\%)=\frac{\mathrm{Wi}-\mathrm{Wd}}{\mathrm{Wd}}\times 100 \qquad (4)$$

Equation S3

Formula (5) was used to calculate the ZOI on each bacterium.

$$\mathrm{ZOI}(\%)=\frac{(\mathrm{D1}-\mathrm{D0})}{\mathrm{D0}}\times 100 \qquad (5)$$

In the formula, D0 is the diameter of inhibition zone in the control group, mm; D1 is the diameter of the inhibition zone in the treatment group, mm.

Equation S4

Percentage of wound closure was analyzed using the following equation (6).

$$\text{Wound closure}(\%) = \frac{\text{Original wound area} - \text{Specific day wound area}}{\text{Original wound area}} \times 100 \quad (6)$$

1.11 Acute toxicity studies

The acute toxicity study of FLL-E followed the Organization for Economic Cooperation and Development (OECD) guideline NO: 401. Twenty animals were randomly assigned to two groups, each containing ten animals. Group I served as the control, while Group II received FLL-E via gavage at a dosage of 2000 mg/kg body weight. Observations were conducted over 14 days to assess alterations in fur, behavior, and any toxic reactions.

1.12 Acute oral toxicity results of FLL-E

As per OECD 401 guidelines, an acute oral toxicity test was conducted using a limit test at a dosage of 2000 mg/kg. This limit test is employed when prior information suggests the test material is expected to have low toxicity, below regulatory limits. Literature findings on the toxicity of PVA and pectin, similar compounds, were considered in this assessment.[7,8] FLL-E exhibited safety at the 2000 mg/kg dosage, showing no observable changes in fur, behavior, or adverse reactions in treated animals. Neither group displayed mortality, and all animals remained active and exhibited normal behavior throughout the observation period.

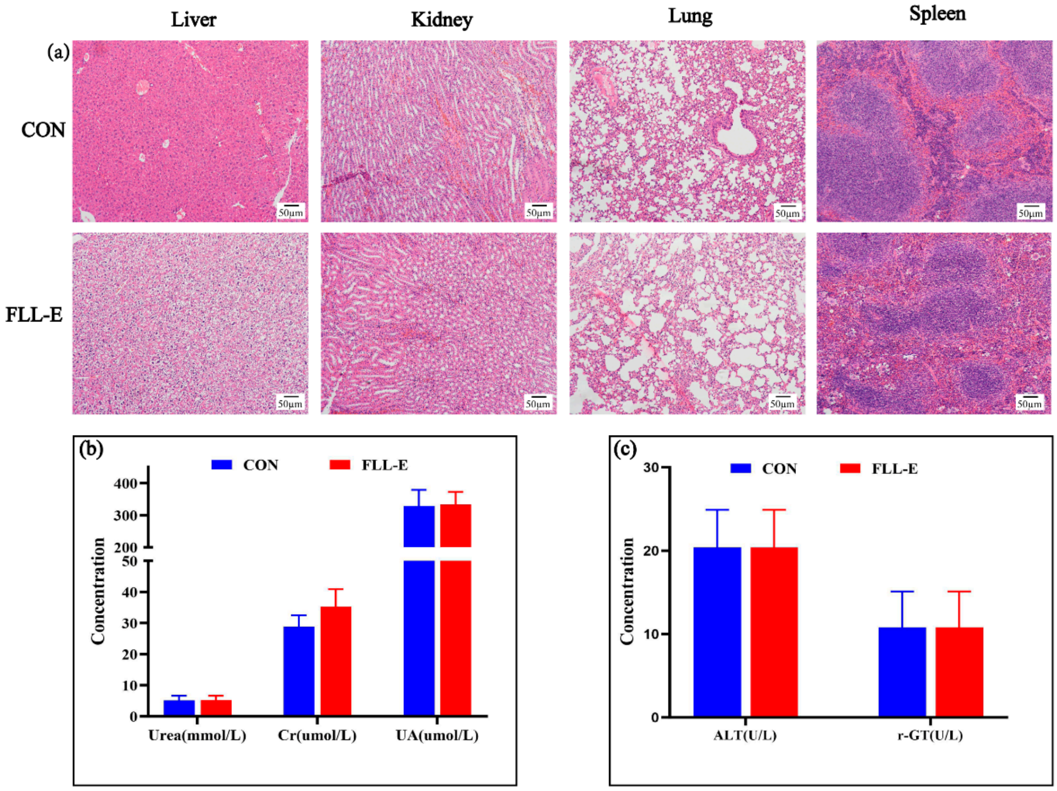

Figure S10. *In vivo* biocompatibility assessment of FLL-E. (**a**) In vivo biocompatibility assessment of FLL-E by major organs H&E sections (Scale bar: 100μm). (**b**) Serum levels of kidney function indicators: blood urea nitrogen (Ure), creatinine (Cr) and uric acid (UA); (**c**) Serum levels of liver function indicators: alanine transaminase (ALT)

and r-glutamyl transpeptidase (r-GT). Error bars indicate SD. Scale bars 50 μm. The magnification is×100.

References

1. Yun, L.; Li, D.; Yang, L.; Zhang, M. Hot water extraction and artificial simulated gastrointestinal digestion of wheat germ polysaccharide. Int. J. Biol. Macromol. 2019, 123, 174–181.
2. Tabara, A.; Oneda, H.; Murayama, R.; Matsui, Y.; Hirano, A.; Seguchi, M. Determination of hydrophobicity of dry-heated wheat starch granules using sucrose fatty acid esters (SFAE). Biosci. Biotechnol. Biochem. 2014, 78, 1572–1576.
3. Ahuja, G, Pathak, K. Porous carriers for controlled/modulated drug delivery. Indian J. Pharm. Sci. 2009, 71, 599-607.
4. Chou, C.T.; Shi, S.C.; Chen, C.K. Sandwich-Structured, Hydrophobic, Nanocellulose-Reinforced Polyvinyl Alcohol as an Alternative Straw Material. Polymers 2021, 13, 4447.
5. Ngo, T.M.; Nguyen, T.H.; Dang, T.M.; Tran, T.X.; Rachtanapun, P. Characteristics and Antimicrobial Properties of Active Edible Films Based on Pectin and Nanochitosan. Int. J. Mol. Sci. 2020, 21, 2224.
6.Fathollahipour, S.; Koosha, M.; Tavakoli, J.; Maziarfar, S.; Mehrabadi, J.F. Erythromycin Releasing PVA/sucrose and PVA/honey Hydrogels as Wound Dressings with Antibacterial Activity and Enhanced Bio-adhesion. Iran. J. Pharm. Res. 2020, 19, 448–464.
7. Irvin, C.W.; Satam, C.C.; Liao, J.; Russo, P.S.; Breedveld, V.; Meredith, J.C.; Shofner, M.L. Synergistic Reinforcement of Composite Hydrogels with Nanofiber Mixtures of Cellulose Nanocrystals and Chitin Nanofibers. Biomacromolecules 2020, 22, 340–352.
8. Riyamol; Gada Chengaiyan, J.; Rana, S.S.; Ahmad, F.; Haque, S.; Capanoglu, E. Recent Advances in the Extraction of Pectin from Various Sources and Industrial Applications. ACS Omega 2023, 8, 46309–46324.
